# Microbatch under-oil salt screening of organic cations: single-crystal growth of active pharmaceutical ingredients

**DOI:** 10.1107/S2052252518017876

**Published:** 2019-01-01

**Authors:** Martin Babor, Philipp P. Nievergelt, Jan Čejka, Vít Zvoníček, Bernhard Spingler

**Affiliations:** a Department of Solid State Chemistry, University of Chemistry and Technology, Prague, Technická 5, Prague 6 166 28, Czech Republic; b Department of Chemistry, University of Zurich, Winterthurerstr. 190, Zurich 8057, Switzerland; c Zentiva k.s., U kabelovny 130, Prague 10 10237, Czech Republic; d Department of Chemical Engineering, University of Chemistry and Technology, Prague, Technická 5, Prague 6 166 28, Czech Republic

**Keywords:** crystallization, active pharmaceutical ingredients, API, polymorphs, small molecules

## Abstract

Multicomponent solid forms of active pharmaceutical ingredients represent a modern method of tuning their physicochemical properties. Typically, salts are the most commonly used multicomponent solid form in the pharmaceutical industry. More than 38% are formulated as organic cations. Salt screening is an essential but demanding step when identifying the most appropriate formulation. The microbatch under-oil crystallization technique of proteins has been combined with the previously developed high-throughput vapour-diffusion screening for use as a novel method of primary salt screening of organic cations. The procedure allows the set up of about 100 crystallization experiments per 30 min. This requires between 17 and 564 mg of screened cationic active pharmaceutical ingredients, which were of moderate to very high water solublity. Five distinct organic salts, three of them diverse active pharmaceutical compounds or the other enantiomer thereof, in the form of chloride salts were tested. The screening was extremely successful; at least two new single-crystal structures could be obtained for each particular compound and many more salts as single crystals were formed compared with our previous vapour-diffusion method.

## Abstract 2

The under-oil crystallization method was successfully adopted for the crystallization of organic salts. Compared with the previously reported vapour-diffusion-based nano-crystallization technique, the under-oil method generated single crystals of more different salts for all studied compounds.

## 1. Introduction   

Control over the multicomponent solid form of an active pharmaceutical ingredient (API) is a modern way of improving physicochemical properties of the API without changing its core chemical structure (Schultheiss & Newman, 2009 ▸). Nowadays, pharmaceutical salts are the most frequently used in multicomponent form in a final drug formulation, corresponding to about 50% of all solid APIs. Positively charged APIs occur in 38% of those FDA approved drugs (prior to the end of 2006), in which the drug has a molecular weight less than 1000 Da (Paulekuhn *et al.*, 2007 ▸). The widely used chloride is increasingly replaced with other anions (Paulekuhn *et al.*, 2007 ▸). The motivation for this development is based on, for example, lower hygroscopicity or targeting that part of the gastrointestinal tract where the salt will be the most soluble (Berge *et al.*, 1977 ▸). The choice of salt is a standard part of any preformulation study (Morissette *et al.*, 2004 ▸). Furthermore, salts other than chlorides can be helpful during the purification of a product.

The standard salt screenings are time, material and labour intensive. New forms are normally generated by direct ionization of the APIs (Morissette *et al.*, 2004 ▸; Tamura *et al.*, 2018 ▸). Surprisingly, to the best of our knowledge, only one method of high-throughput salt screening using ion exchange has been published (Nievergelt *et al.*, 2018 ▸). It is a modification of the vapour-diffusion crystallization method (VDHT) originally developed and optimized for macromolecules (McPherson & Gavira, 2014 ▸). In the method developed by us (Nievergelt *et al.*, 2018 ▸), a water-soluble organic cationic salt (normally in chloride form) is mixed with a water-soluble sodium or potassium salt to generate a new, less water-soluble salt of the organic cation with a new anion. Another feasible modification of the crystallization procedure is microbatch under-oil crystallization (Chayen *et al.*, 1992 ▸). The biggest advantage of under-oil crystallization compared with the vapour-diffusion method is the higher level of concentration that can be achieved (see Fig. S1 of the supporting information). The vapour-diffusion method achieves supersaturation by equalization of the water vapour pressure between two different solutions. The first solution (the drop in which the crystallization shall take place) is normally a 1:1 mixture of the stock solution of the screened cationic API and the stock solution of the anion. The second one is a pure stock solution of the anion. The water activity in the drop is approximately twice that of the anion stock solution, as this solution has been one-time diluted by addition of the aqueous solution of the organic cation. Having a small drop and a huge reservoir with the stock solution of the anion, which both share the same vapour phase, the final concentration of the organic cation within the drop will reach approximately twice the starting concentration. In contrast, under-oil screening achieves supersaturation by slow penetration of water through the silicone oil (Fig. S1 of the supporting information). Hence, the screened drop is slowly concentrated until an almost dry residue is obtained. Moreover, microbatch under-oil crystallization can be performed with standard laboratory equipment such as multichannel pipettes and crystallization plates without the need for pipetting robots. On the other hand, one could use a robot for setting up the crystallization plates as has already been done in the field of protein crystallization (Chayen *et al.*, 1990 ▸; McPherson & Gavira, 2014 ▸). Furthermore, using different vessels, this method might also be upscalable. Note that the probability of crystallizing one of the starting materials during under-oil crystallization is higher, rendering the evaluation more difficult (Baldock *et al.*, 1996 ▸).

**Figure 1:**
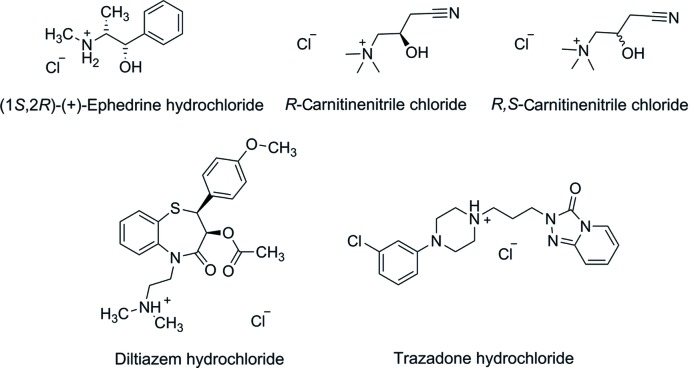
Chemical structures of the screened organic cations in the form of chlorides or hydro­chlorides.

In this work, we investigated the crystallization of five organic cations (Fig. 1 ▸), two of which are APIs. *R*,*S*-carnitine­nitrile chloride and *R*-carnitine­nitrile chloride {[(±)-Car]Cl and [(−)-Car]Cl} are precursors in the synthesis of carnitine or its derivatives. Carnitine is used in the treatment of different diseases from neurological problems to diabetes mellitus. Only the crystal structure of the tetra­phenyl­borate salts of [(±)-Car]^+^ and [(−)-Car]^+^ have been reported previously (Nievergelt *et al.*, 2018 ▸). (1*S*,2*R*)-(+)-Ephedrine hydro­chloride ([(+)-EphH]Cl) is the other enantiomer of the naturally occurring (1*R*,2*S*)-(−)-ephedrine, which is employed for the treatment of bronchial asthma and emphysema. (1*S*,2*R*)-(+)-Ephedrine is, in general, pharmacologically inactive compared with its other enantiomer (Lee, 2011 ▸). There are many structures of salts of the various diastereomers of ephedrine (Collier *et al.*, 2006 ▸; Wu *et al.*, 2012 ▸) described in the Cambridge Structural Database (Groom *et al.*, 2016 ▸). Diltiazem hydro­chloride ([DilH]Cl) is a well known calcium channel blocking agent, which is used for the treatment of stable angina pectoris and hypertension. Five crystal structures containing diltiazem have been reported and four of them are salts (Kojić-Prodić *et al.*, 1984 ▸; Tanaka *et al.*, 1992 ▸; Stepanovs *et al.*, 2016 ▸). Trazodone hydro­chloride ([TrH]Cl) is being used pharmaceutically as an antidepressant (Davidoff *et al.*, 1987 ▸). The crystal structures of the protonated trazodone chloride (Fillers & Hawkinson, 1979 ▸), iodide and oxalate (Nievergelt *et al.*, 2018 ▸) salts have been reported previously.

## 2. Experimental   

(1*S*,2*R*)-(+)-Ephedrine hydro­chloride and trazodone hydro­chloride were obtained from Sigma–Aldrich. *R*-Carnitine­nitrile chloride was obtained from Angene Chemical, Hong Kong, HK. *R*,*S*-Carnitine­nitrile chloride was obtained from Frontier Scientific, Logan, UT, USA. Diltiazem hydro­chloride was provided by Zentiva k.s., Prague, CZ. The used silicone oil (unless otherwise noted) was article number 146153 from Sigma–Aldrich; it has a viscosity of 50 cSt (1 cSt = 1 mm^2^ s^−1^) and is normally used for melting-point and boiling-point apparatus. This oil was previously used in under-oil screenings of proteins (Vetting *et al.*, 2009 ▸). For a few experiments, a silicone oil with a much lower viscosity of 5 cSt was employed (article number: 317667 from Sigma–Aldrich). Sodium or potassium salts of suitable counterions were obtained from various commercial suppliers. The reason for choosing either a sodium or potassium salt was based on the accessibility of one versus the other (*e.g.* sodium hydrogen phthalate is not commercially available, however, its potassium salt is). In cases when the sodium or potassium salts were not commercially available, the sodium salts were prepared by titration of the corresponding acids with a sodium hydroxide solution (2*M*) until a pH of 7 was reached. The solutions of the newly prepared salts were concentrated with the help of a rotary evaporator and dried by lyophilization. The water content was determined by elemental analysis (carbon, hydrogen and nitro­gen) of each new salt. The concentrations of the anion solutions were chosen such that they were about half saturated. In the case of high concentrations, lower concentrations of the same anion were also employed in order to test the influence of the concentration of the anions.

The screening technique described in this publication combines the primary salt screening of APIs and growing single crystals of hits. Silicone oil (100 µl) was added to each well in a 96 round bottom well costar 3795 plate (Corning Incorporated, USA). Stock solutions of four organic cations including two APIs [diltiazem hydro­chloride, (*R*,*S*)-carnitine­nitrile chloride, (*R*)-carnitine­nitrile chloride, (1*S*,2*R*)-(+)-ephedrine hydro­chloride, 5 µl each; 90% maximal saturation in water] were pipetted with the help of an eight-channel pipette directly into the silicone oil within the wells. The drops of these aqueous solutions sank to the bottom of the well. Afterwards, individual stock solutions of the counterions (5 µl) were added to each well with silicone oil and the to-be-crystallized organic cation. When trying different volumes of solutions, volumes smaller than 5 µl of the concentrated anion solutions were difficult to pipette due to their high viscosity, yet volumes larger than 5 µl of the analyte were actually superfluous and wasted stock solutions. In some cases, it was necessary to combine two separate aqueous drops with a pipette tip under oil in order to have one common drop. In every well, there must be just one drop of the mixture, which is crucial for the under-oil experiment. Initial screenings were carried out for 147 different conditions with 86 different counterions (Table S1).

In a second series of experiments, we concentrated on ephedrine and trazodone as well as on 96 promising anion solutions (see Table S2). Ephedrine is known to form many salts (Collier *et al.*, 2006 ▸), some of which we did not obtain with our first series of experiments. Secondly, all studied cations of the first series were highly soluble or even extremely soluble in water. In order to have a compound that also displays moderate but not too high solubility in water, we selected trazodone hydro­chloride, which we had studied in our previous vapour-diffusion investigation (Nievergelt *et al.*, 2018 ▸). Compared with the 147 conditions used previously, we eliminated anions that were only soluble at millimolar concentrations (*e.g.* sodium do­decyl­sulfate) or whose solution became black because of lack of chemical stability (sodium 4-amino­salicylate). Furthermore, the chosen maximum concentration of the anion salt should approximately correspond to a half-saturated solution of the salt of that very anion. Therefore, some of the initial concentrations of the sodium or potassium salts were increased as we discovered that these concentrations had been well below half-saturation in our first series of experiments. Additionally, we used smaller volumes of the organic cation solution compared with the volumes of the anion solutions in order to promote anion exchange. For ephedrine, we mixed 2 µl of 90% saturated ephedrine solution and 20 µl of counterion solution. For trazodone, we mixed 4 µl of 90% saturated trazodone solution and 10 µl of counterion solution. We monitored all wells essentially every day for 30 days and marked the outcome of the crystallizations. Promising looking crystals were isolated and placed in Infineum V8512 oil as soon as possible as the solutions continued to become more concentrated. In some cases, it was necessary to induce crystal growth by scratching the wells with a dissecting needle.

Single-crystal X-ray diffraction patterns were measured on a Rigaku-Oxford Diffraction XtaLAB Synergy-S dual source diffractometer: Kappa-axis four-circle goniometer with a Dectris Pilatus3 R 200 K hybrid pixel area detector and Cu and Mo PhotonJet microfocus X-ray sources. The data collection strategy and data reduction were performed using *CrysAlisPRO* (Rigaku Oxford Diffraction, 2015 ▸). Crystals were fished out using a micro-spoon spatula from Bochem (35781 Weilburg, Germany, article number 3344) also available at VWR (article number 231–1355). The crystals were prepared on a glass slide under Infineum V8512 oil and the single crystals were mounted on top of a 18 mm Mounted CryoLoop in a CrystalCap Magnetic (Hampton Research). The structures were solved using *SUPERFLIP* (Palatinus & Chapuis, 2007 ▸), *SIR92* (Altomare *et al.*, 1994 ▸) or *SHELXT* (Sheldrick, 2015*a* ▸), and were refined in *CRYSTALS* (Betteridge *et al.*, 2003 ▸) or *SHELXL2014* (Sheldrick, 2015*b* ▸). Graphical output was made with the help of *Mercury* (Macrae *et al.*, 2008 ▸).

## 3. Results and discussion   

Protein micro-batch crystallization (Chayen *et al.*, 1992 ▸) was modified to be used for the salt screening of organic cations. Initially, we chose four different organic cations for the screening, two permanent cations [(*R*,*S*)-carnitine­nitrile chloride and (*R*)-carnitine­nitrile chloride] and two bases as their hydro­chloride salts [diltiazem hydro­chloride and (1*S*,2*R*)-(+)-ephedrine hydro­chloride]. Their approximate solubilities are given in Table 1 ▸. Initial screenings were carried out for 147 different conditions with 86 different counterions (Table S1). The chosen anions were selected to include many diverse inorganic and organic anions, many of them fulfilling the GRAS condition (Select Committee on GRAS Substances, 2017 ▸), some of them were selected because of their propensity to form crystalline salts. As we have found previously (Nievergelt *et al.*, 2018 ▸) that half-saturated anion solutions work well, we also employed them here. The concentration of some selected anion solutions with concentrations higher than 2*M* were halved once or twice in order to promote crystal growth rather than a powdery precipitate. As the initially chosen compounds (carnitine­nitrile chloride, diltiazem and ephedrine hydro­chloride) all exhibited rather high solubility and only formed new crystal salts in the presence of highly concentrated anions, we set up a second round of optimization. First, we changed the volume ratio of cation solution to anion solution in the case of ephedrine from 1:1 to 1:10 in order to promote the anion exchange. Secondly, we selected an additional API, trazodone, that we had already tested in the vapour-diffusion nano-crystallization technique (Nievergelt *et al.*, 2018 ▸) and that has a 10× lower aqueous solubility than ephedrine, the organic salt with the lowest solubility in the current study so far (Table 1 ▸). For trazodone, we chose an API to anion solution volume ratio of 1:2.5. Finally, we reduced the number of crystallization trials per analyte in order to efficiently use 96 well plates (for details, see the *Experimental* [Sec sec2]).

**Figure 2:**
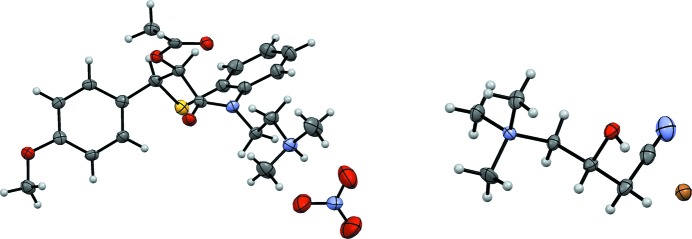
Left: displacement ellipsoid representation of [DilH][NO_3_]. Right: displacement ellipsoid representation of [(−)-Car]Br. Ellipsoids are depicted at 50% probability.

The formation of single crystals of the organic cation together with an anion other than chloride (if present) was selected as the desired endpoint of the crystallization trials. The positive results of the crystallization experiments, including the concentration of the counterion solution used, are summarized in Table 2 ▸. Additionally, the number of the day on which crystals were first observed is given (*e.g.* D0: crystals observed on the day of setting up the experiment). We have succeeded in the crystallization of at least two salts for each screened cation. In three cases, we observed crystallization of a supersaturated solution directly after having touched the drop with a spatula or scratched the well with a dissecting needle. The resulting crystals were the new bromide (see Fig. 2 ▸), tetra­fluoro­borate and iodide salts of [(−)-Car]^+^ as well as the iodide salt of [(±)-Car]^+^. Furthermore, there were three positive hits with too low-quality crystals for structural analysis of [(±)-Car] bromide, [(±)-Car] tetra­phenyl­borate and [(−)-Car] tetra­phenyl­borate. Moreover, we succeeded in the crystallization of four new salt forms of diltiazem (see Fig. 2 ▸). The diltiazem crystal structures of the bromide, the iodide and the nitrate salts are essentially isostructural with the published data on the chloride salt (Kojić-Prodić *et al.*, 1984 ▸). Additionally, we tried to crystallize the pure solution of the screened cations in the forms of chlorides or hydro­chlorides using the under-oil technique. In this way, the unit cells of the crystals of the [DilH]Cl and [(+)-EphH]Cl salts were found to correspond to the known forms of the hydro­chlorides. [(−)-Car]Cl crystallized as high-quality single crystals. Surprisingly, *R*,*S*-carnitine­nitrile chloride crystallized with two *S*-carnitine­nitrile and one *R*-carnitine­nitrile cations in the asymmetric unit of the chiral space group *P*2_1_. Such a rare system has been described in the literature either as a pseudo unbalanced crystallization, co-crystals of a racemate or unbalanced chiral packing (Fábián & Brock, 2010 ▸; Albrecht *et al.*, 2010 ▸; Wachter *et al.*, 2016 ▸; Kotelnikova *et al.*, 2017 ▸; Grothe *et al.*, 2017 ▸). The remaining observed negative crystallization results were either mixtures of NaCl (Fig. S2) and amorphous residue, amorphous residue only or pure chlorides (or hydro­chlorides) of the tested anions.

As a next step for improving the under-oil method, we employed a larger volume of anion solution compared with the analyte solution. This increases the anion to organic cation ratio and therefore was predicted to favour the formation of the new salt. Finally, we reduced the number of anion solutions to just 96 selected conditions (ignoring the salt free condition), allowing one series of screening experiments to be carried out in just one 96 well plate. We eliminated anions with a low millimolar solubility that would not promote a quantitative anion exchange and anions that were determined to be unstable in an aqueous, aerated solution [such as sodium 4-amino­salicylate (*The Merck Index*, 1976 ▸)]. Indeed, the improved method yielded five salts of trazodone, of which three were novel. The second round of crystallization of ephedrine with a 1:10 volumetric ratio of analyte solution versus anion solution gave seven additional salts, of which four were new. One of the novel structures was a new polymorph of ephedrinium nitrate, which crystallized in the chiral space group *P*2_1_ with two formula units in the asymmetric unit and unit-cell dimensions of *a* = 6.0401 (3), *b* = 29.3553 (8), *c* = 7.3828 (3) Å, β = 112.806 (5)° and *V* = 1206.70 (9) Å^3^. Later, three-dimensional crystals (Fig. S15) were produced that were identified as the known nitrate salt polymorph I (Collier *et al.*, 2006 ▸), which also crystallized in the monoclinic space group *P*2_1_, but with just one formula unit in the asymmetric unit and with unit-cell dimensions of *a* = 5.536 (5) Å, *b* = 6.839 (9) Å, *c* = 15.669 (12) Å, β = 97.28 (7)° and *V* = 588 (1) Å^3^. In polymorph I, the ephedrine cation adopts a folded conformation, while in polymorph II it is in an extended conformation. Further anions that formed novel crystal structures with ephedrine, are l-tartrate, which crystallizes as a monohydrate in the chiral space group *P*2_1_. The trihydrate has previously been reported by Collier *et al.* (2006 ▸). Additionally, we succeeded in crystallizing the monohydrate of l-malate; its anhydrate form has been described again by Collier. For trazodone, three novel crystal structures of its protonated form with either nitrate, tetra­fluoro­borate or thio­cyanate could be determined. For all wells in which no crystallization could be observed, the drops were punctured with a spatula or the wells below the drops scratched with a preparation needle. Since in a few cases, crystal growth in some over-saturated drops could be observed after this procedure, we recommend performing this easy step for all wells in which no crystals have formed after a few weeks.

In order to study the influence of the ratio of anion to organic cation, the cation of the salt that provides the final anion and the used oil, we performed detailed design experiments for two selected anions, iodide and oxalate (Tables S3 and S4). For the crystallization of ephedrinium iodide, the ratio of ephedrinium to iodide was varied between 1 and 200. Additionally, lithium, sodium and potassium iodide at the same concentration were compared in order to assess their influence on the crystallization of ephedrinium iodide. And thirdly, the whole series was repeated with a silicone oil possessing a 10× lower viscosity (5 cSt) than the one previously used, as d’Arcy and co-workers have shown that the viscosity of the silicone oil more or less linearly correlates with the time it takes for the protein crystals to appear (D’Arcy *et al.*, 1996 ▸). The summary of our results of the crystallization of ephedrinium iodide (Table S3) is as follows. Crystals were observed within a 5- to 50-fold ratio of iodide to ephedrinium. At a ratio smaller than 5, there seems to be insufficient excess iodide present. At a ratio higher than 50, there was not enough ephedrinium in the drops. While small differences could be noticed, no clear effects of the cation of the iodide salt nor the used silicone oil could be established. For the crystallization of bis-ephedrinium oxalate (Table S4), the influence of the ratio of oxalate to ephedrinium and the used silicone oil was studied. The growth of ephedrinium oxalate crystals could be observed over a wide ratio between 0.01 and 3.3 up to 33.3 (depending on the oil used). If the ratio of oxalate to ephedrinium was 0.1 or lower, after a number of days the remaining ephedrinium chloride formed large crystals in both oils.

As a final step, we compared the results of the under-oil microbatch screening with the VDHT technique (Nievergelt *et al.*, 2018 ▸). The chosen cations for the comparison were [(−)-Car]^+^, [(±)-Car]^+^, [(+)-EphH]^+^ and [TrazH]^+^ with decreasing solubilities in this order (see Tables 1 ▸ and 3 ▸). The under-oil microbatch screening was able to produce many more crystalline salts with an exchanged anion than with the VDHT screening for any of the four compared cations (see Table 3 ▸). The reason for the superior performance of the under-oil method most likely lies in the higher supersaturation that can be achieved with the under-oil method compared with vapour diffusion.

When analysing the results of our crystallizations, it became clear that mainly anions that were present in equally high or higher concentrations than the organic cations, crystallized together with them. The only anion that does not follow this rule is oxalate. This is in accordance with a report by Stepanovs and co-workers, who described a system also containing a methyl­ammonium ethanol unit. The oxalate salt of propranolol had a 28-fold lower aqueous solubility of the organic cation when compared with the chloride salt (Stepanovs *et al.*, 2015 ▸). Additionally, we compared the success rate of the under-oil technique with the classical titration method. Davey and co-workers (Collier *et al.*, 2006 ▸) report the synthesis and crystallization of 16 salt forms starting from the free base ephedrine and adding one or half of an equivalent of acid or diacid. Three different solvents were employed for the synthesis and subsequent crystal growth. Our direct approach with the optimized procedure starting from one single solution of ephedrinium chloride directly yielded single crystals of eleven salt forms apart from the starting chloride salt. Four of them had novel crystal structures, including one new polymorph of ephedrinium nitrate.

## 4. Conclusions   

The under-oil crystallization technique for proteins was successfully adapted for use in salt screening of APIs. After an optimization procedure, the ideal screening involves 96 crystallization batches that consume less than 200 µl of a 90% saturated solution for each screened API and the experiment can be set up within *ca* 30 min. For each screened cation, we could determine the single-crystal structure of at least two new salts, each one with a different anion. Five salts of [DilH]^+^ and twelve salts of [(+)-EphH]^+^ were observed and their crystals grew in sufficient quality for single-crystal X-ray determination (SCXRD). Two of the four observed crystals of salts of [(±)-Car]^+^ and four of the five of [(−)-Car]^+^ crystallized in sufficient quality for SCXRD. To the best of our knowledge, this is the first application of the microbatch under-oil crystallization technique for the crystal growth of small molecules. Finally, the under-oil screening was compared to the vapour-diffusion method. The under-oil method was found to be much more effective in generating single crystals for all five compounds, which were crystallized by both methods (Table 3 ▸). Additionally, the under-oil technique does not require the use of an expensive pipetting robot. In total, 17 new salts of the studied five cations were prepared. On the other hand, the under-oil technique consumes a little more material because the individual experiment requires a higher volume of analyte solution of between 2 and 5 µl rather than 100 nl in the vapour-diffusion experiment performed by the liquid handling robot. As a final remark, we note that the under-oil technique favours the crystallization of any kind of water-soluble substance from aqueous solutions.

## 5. Related literature   

The following references are cited in the supporting information: Prince (1982 ▸); Watkin (1994 ▸).

## Supplementary Material

Crystal structure: contains datablock(s) Underoil, RScarnitinenitrilechloride, RScarnitinenitrileiodide, Rcarnitinenitrilechloride, Rcarnitinenitrilebromide, Rcarnitinenitrileiodide, Rcarnitinenitrile2tetrafluoroborate1chloride1, Diltiazembromide, Diltiazemiodide, Diltiazemnitrate, Diltiazemphosphatesesquihydrate, pn180815_uo_eph_1_180814_pyrolidcarbox, pn180815_uo_traz_1_180814_c6_nitrate_mo, pn180815_uo_traz_1_180814_scn, pn180817_uo_trh_1_180814_b1_bf4, pn180830_uo_eph_1_180814_b12_benzsulf, pn180914_uo_eph_1_180814_g4_2, pn180914_uo_eph_1_180814_g5_tart, pn180914_uo_eph_2_180814_c6_scr2. DOI: 10.1107/S2052252518017876/lt5016sup1.cif

Supporting information with general methods, figures and (crystallographic) tables. DOI: 10.1107/S2052252518017876/lt5016sup2.pdf

CCDC references: 1860743, 1860744, 1860745, 1860746, 1860747, 1860748, 1860749, 1860750, 1860751, 1860752, 1885877, 1885878, 1885879, 1885880, 1885881, 1885882, 1885883, 1885884

## Figures and Tables

**Table 1:** Screened cations and their aqueous solubility at 22°C

	Approximate solubility (mg ml^−1^)	Amount needed (mg)†	Solution molarity [*M*]‡
Diltiazem hydro­chloride	720 ± 70§	312	1.44
(*R*,*S*)-Carnitine­nitrile chloride	1120 ± 106¶	486	5.64
(*R*)-Carnitine­nitrile chloride	1300 ± 200¶	564	6.54
(1*S*,2*R*)-(+)-Ephedrine hydro­chloride	257± 1¶	111	1.15
Trazodone hydro­chloride	38.5 ± 0.2¶	17	0.085

† For screening 97 conditions.

‡ At 90% saturation.

§ Determined as described by Nievergelt *et al.* (2018 ▸).

¶ Value taken from the work by Nievergelt *et al.* (2018 ▸).

**Table 2:** Obtained single crystals Crystals were grown by mixing 5 µl of the organic cation solution and 5 µl of the anion solution unless otherwise noted. DX: crystals were observed after X days.

Salt providing the new anion	Molarity	[(+)-EphH]Cl	[(−)-Car]Cl	[(±)-Car]Cl	[DilH]Cl	[TrazH]Cl
Sodium bromide	4.08	D0†	D14‡		D5	
Sodium iodide	5.34	D5†	D8‡	D11	D6	D1†, §
Sodium tetra­fluoro­borate	2.00		D10¶			D3§
Potassium thio­cyanate	7.34	D2†				D1§
Sodium nitrate	4.61	D30‡, ††, [Table-fn tfn10]			D6	D1§
Sodium di­hydrogen phosphate	3.40				D5	
Sodium pyrrolidone carboxyl­ate	4.96	D1††				
Sodium benzene­sulfonate	1.10	D16†, ††				
Disodium oxalate	0.138	D6†, ††				D1†, §
Disodium malonate	2.97	D16†, ††				
Sodium L-malate	2.92	D30††				
Potassium sodium L-tartrate	1.40	D30††				
No additional salt added		D15†	D14	D7	D6†	

† Published structure, sometimes of the other enantiomer (Hearn & Bugg, 1972 ▸; Kojić-Prodić *et al.*, 1984 ▸; Collier *et al.*, 2006 ▸; Wu *et al.*, 2012 ▸; Nievergelt *et al.*, 2018 ▸).

‡ Crystallized after touching the drop with a spatula or scratching the well with a dissecting needle.

§ Obtained by mixing 4 µl of the trazodone solution and 10 µl of the anion solution.

¶ Concentration of sodium tetra­fluoro­borate was 4.0*M*.

†† Obtained by mixing 2 µl of the ephedrine solution and 20 µl of the anion solution.

‡‡ Two polymorphs (I† and II) observed.

**Table 3:** Comparison of the results obtained by under-oil and vapour-diffusion (VDHT) methods (×): no salt crystal was obtained during the screening. Abbreviations: tetra­phenyl­borate: [TPB]^−^; thio­cyanate: [SCN]^−^; oxalate: [OXA]^2−^; benzene­sulfonate [BS]^−^; pyrrolidone carboxyl­ate: [Pyrcarb]^−^.

Cation to be crystallized	[(+)-EphH]^+^		[(−)-Car]^+^		[(±)-Car]^+^		[TrazH]Cl	
Method	Under oil	VDHT	Under oil	VDHT	Under oil	VDHT	Under oil	VDHT
Anions	Cl^−^	Cl^−^	[TPB]^−^	[TPB]^−^	[TPB]^−^	[TPB]^−^	I^−^	I^−^
	Br^−^	Br^−^	Cl^−^	×	Cl^−^	×	[OXA]^2−^	[OXA]^2−^
	I^−^	I^−^	Br^−^	×	Br^−^	×	NO_3_^−^	×
	[SCN]^−^	[SCN]^−^	I^−^	×	I^−^	×	[BF_4_]^−^	×
	[OXA]^2−^	[OXA]^2−^	[BF_4_]^−^:Cl^−^	×			[SCN]^−^	×
	NO_3_^− ^ †	×						
	[BS]^−^	×						
	[Malonate]^2−^	×						
	[L-Malate]^2−^	×						
	[L-Tartrate]^2−^	×						
	[Pyrcarb]^−^	×						

† Two polymorphs obtained.
