# Supplementary material for: Microbatch under-oil salt screening of organic cations: single-crystal growth of active pharmaceutical ingredients
Source: IUCrJ. 2019 Jan 1;6(Pt 1):145–51. doi: 10.1107/S2052252518017876 (PMC6327184; doi:10.1107/S2052252518017876)
Supplement: Supplementary file 2 [file m-06-00145-sup2.pdf]

IUCrJ

Volume 6 (2019)

Supporting information for article:

Microbatch under-oil salt screening of organic cations: single-crystal growth of active pharmaceutical ingredients

Martin Babor, Philipp P. Nievergelt, Jan Čejka, Vít Zvoníček and Bernhard Spingler

S1. General methods

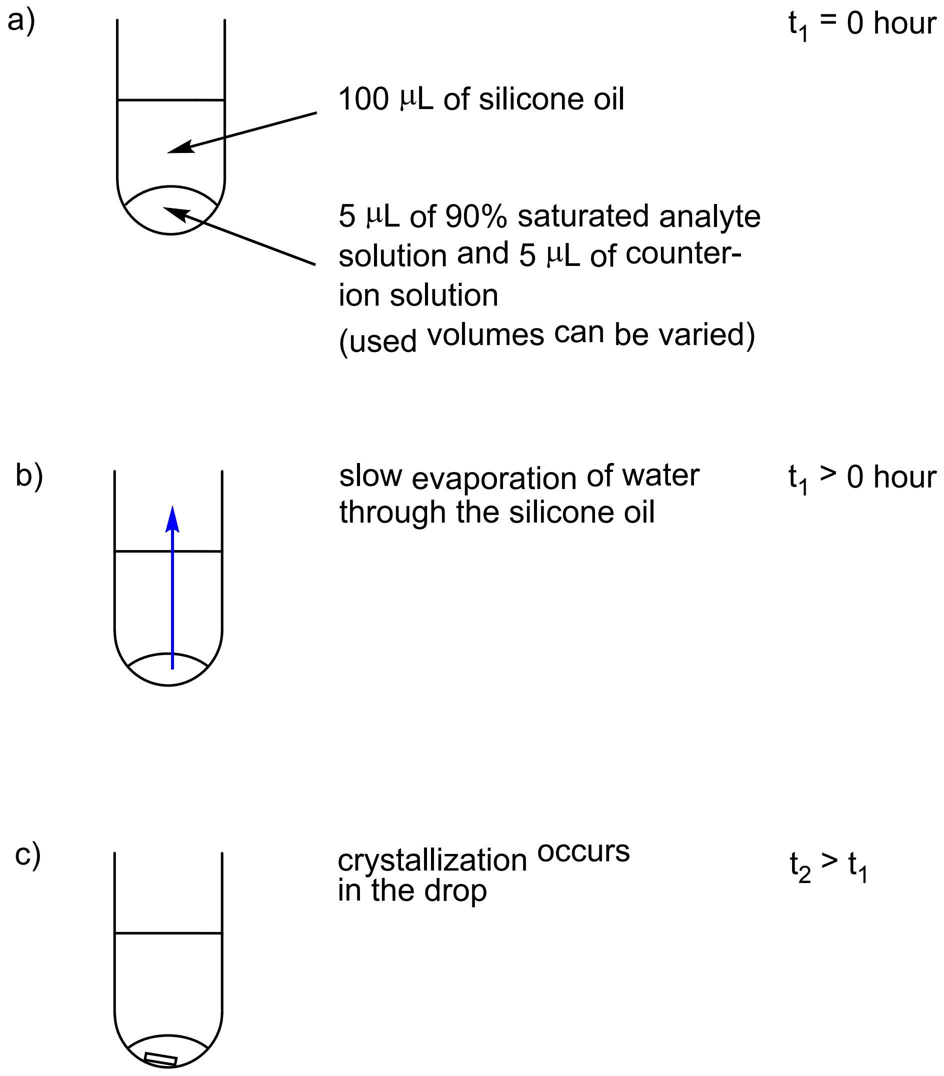

Figure S1 Principle of the microbatch under oil crystallization technique

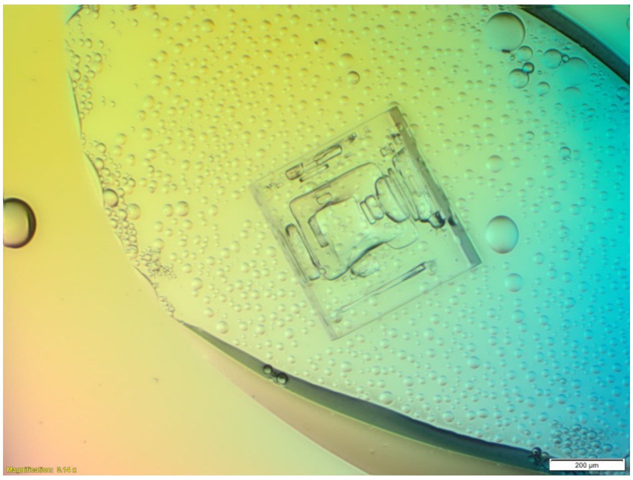

Figure S2 Example of the mixture of NaCl (in the middle) and amorphous residuum (around NaCl)

Table S1 List of originally used anion sources and their respective salt concentrations (147 conditions)

Salts	Molarity	Salts	Molarity
Sodium bromide	4.082	Sodium *D*-mandelate	0.251
Sodium bromide	2.000	Sodium *D*-mandelate	0.100
Sodium iodide	5.337	Sodium *L*-mandelate	0.251
Sodium iodide	3.000	Disodium succinate	0.391
Sodium tetrafluoroborate	4.076	Disodium succinate	0.200
Sodium tetrafluoroborate	2.000	Sodium *DL*-aspartate	0.250
Sodium methanesulfonate	3.651	Sodium *DL*-aspartate	0.100
Sodium methanesulfonate	1.700	Sodium *L*-aspartate	0.244
Sodium triflate	0.819	Sodium *L*-aspartate	0.100
Sodium triflate	0.400	Disodium *DL*-tartrate	0.282
Potassium hexafluorophosphate	0.244	Disodium *DL*-tartrate	0.150
Potassium hexafluorophosphate	0.100	Disodium (+)-O,O'-dibenzoyl-*D*-tartrate	0.022
Sodium 3-nitrobenzensulfonate	0.423	Sodium N-acetylglycinate	0.178
Sodium 3-nitrobenzensulfonate	0.200	Sodium N-acetylglycinate	0.100
Potassium thiocyanate	7.341	Disodium maleate	0.126

Potassium thiocyanate	3.500	Sodium pyrrolidone carboxylate	0.531
Sodium nitrate	4.605	Sodium pyrrolidone carboxylate	0.250
Sodium nitrate	2.000	Disodium N-acetylglutamate	0.572
Sodium benzoate	1.817	Disodium N-acetylglutamate	0.250
Sodium benzoate	0.800	Disodium *DL*-malate	1.051
Potassium phthalate monobasic	0.271	Disodium *DL*-malate	0.500
Potassium phthalate monobasic	0.150	Sodium *p*-toluenesulfonate	0.153
Sodium formate	6.002	Sodium camphorsulfonate	0.291
Sodium formate	3.000	Sodium camphorsulfonate	0.150
Sodium acetate	2.577	Trisodium citrate	0.050
Sodium acetate	1.200	Trisodium citrate	0.025
Sodium trifluoroacetate	2.401	Disodium pamoate	0.124
Sodium trifluoroacetate	1.000	Disodium pamoate	0.050
Sodium iodoacetate	0.244	Disodium fumarate	0.688
Sodium hippurate	0.244	Disodium fumarate	0.350
Sodium potassium *L*-tartrate	1.050	Sodium propionate	3.984
Sodium potassium *L*-tartrate	0.500	Sodium propionate	2.000
Potassium antimony tartrate	0.041	Potassium *D*-saccharate	0.045
Sodium diethyldithiocarbamate	0.011	Potassium *D*-saccharate	0.020
Disodium malonate	0.488	Sodium valerate	0.097
Disodium malonate	0.250	Sodium valerate	0.050
Sodium pyruvate	2.980	Disodium hydrogen phosphate	0.428
Sodium pyruvate	1.500	Disodium hydrogen phosphate	0.200
Sodium salicylate	2.227	Sodium dihydrogen phosphate	3.399
Sodium salicylate	1.000	Sodium dihydrogen phosphate	1.700
Sodium 2-ethylhexanoate	2.111	Sodium chloride	2.942
Sodium 2-ethylhexanoate	1.000	Sodium chloride	1.500
Sodium *L*-glutamate	1.986	Sodium isethionate	1.908
Sodium *L*-glutamate	0.900	Sodium isethionate	1.000

Sodium sulphate	0.955	Disodium oxalate	0.138
Sodium sulphate	0.500	Disodium oxalate	0.075
Sodium tetraphenylborate	0.409	Sodium hydrogen carbonate	0.564
Sodium tetraphenylborate	0.200	Sodium hydrogen carbonate	0.300
Sodium dodecylsulfate	0.007	Sodium benzenesulfonate	0.130
Sodium diclofenac	0.002	Sodium benzenesulfonate	0.075
Potassium gluconate	1.095	Sodium hexanoate	1.955
Potassium gluconate	0.500	Sodium hexanoate	1.000
Sodium saccharine	0.238	Disodium carbonate	1.014
Sodium 1-naphthalenacetate	0.428	Disodium carbonate	0.500
Sodium 1-naphthalenacetate	0.200	Sodium 4-aminosalicylate	1.488
Sodium 1-naphthalensulfonate	0.346	Sodium 4-aminosalicylate	0.750
Sodium 1-naphthalensulfonate	0.150	Sodium propionate	1.000
Sodium 2-naphthalensulfonate	0.127	Sodium propionate	0.500
Disodium 2, 6-naphthalenedisulfonate	0.084	Sodium dihydrogen phosphate	0.850
Sodium *DL*-lactate	1.450	Sodium chloride	0.750
Sodium *DL*-lactate	0.600	Sodium isethionate	0.500
Sodium *L*-lactate	1.450	Sodium hexanoate	0.500
Sodium *L*-lactate	0.600	Disodium carbonate	0.250
Sodium diphenylacetate	0.329	Sodium 4-aminosalicylate	0.375
Sodium diphenylacetate	0.100	Sodium adipate	0.630
Disodium isophthalate	0.061	Sodium *L*-malate	1.710
Disodium terephthalate	0.060	Sodium dicyanamide	0.700
Sodium meta-hydroxybenzoate	1.315	Sodium nicotinate	2.288
Sodium meta-hydroxybenzoate	0.600	Sodium nicotinate	1.125
Sodium *DL*-mandelate	0.251	Sodium octanoate	0.900
Sodium *DL*-mandelate	0.100	Disodium 1,5-Naphthalenedisulfonate	0.180
Potassium sorbate	2.079	Sodium cinnamate	0.180
Sodium cholate	0.933	Disodium glutarate	0.965

Salt free well

Table S2 List of selected anion sources and their respective salt concentrations (97 conditions)

Salts	Molarity	Salts	Molarity
Sodium fluoride	0.50	Sodium *DL*-lactate	3.42
Sodium chloride	3.00	Sodium *L*-lactate	3.42
Sodium chloride	1.50	Sodium diphenylacetate	0.33
Sodium bromide	4.00	Disodium isophthalate	1.40
Sodium bromide	2.00	Disodium terephthalate	0.060
Sodium iodide	5.30	Sodium meta-hydroxybenzoate	2.90
Sodium iodide	2.50	Sodium *DL*-mandelate	0.25
Sodium iodide	1.25	Sodium *D*-mandelate	0.25
Sodium tetrafluoroborate	4.00	Sodium *L*-mandelate	0.25
Sodium tetrafluoroborate	2.00	Sodium 2-phenylproprionate	1.70
Sodium methanesulfonate	3.60	Disodium succinate	1.13
Sodium methanesulfonate	1.80	Potassium *DL*-aspartate	0.25
Sodium triflate	0.80	Sodium *L*-aspartate	0.25
Potassium hexafluorophosphate	0.24	Disodium *DL*-tartrate	0.55
Sodium 3-nitrobenzensulfonate	0.42	Disodium *L*-tartrate	1.00
Potassium thiocyanate	7.30	Disodium (+)-O,O'-dibenzoyl-*D*-tartrate	0.26
Potassium thiocyanate	3.50	Sodium N-acetylglycinate	2.28
Potassium thiocyanate	1.75	Disodium maleate	0.66
Sodium nitrate	4.60	Sodium pyrrolidone carboxylate	4.96
Sodium nitrate	2.30	Disodium N-acetylglutamate	1.63
Sodium benzoate	1.80	Disodium *DL*-malate	2.27
Potassium phthalate monobasic	0.27	Sodium *p*-toluenesulfonate	0.70
Sodium formate	6.00	Sodium camphorsulfonate	2.28
Sodium formate	3.00	Trisodium citrate	1.20

Sodium acetate	2.60	Disodium citrate	0.96
Sodium trifluoroacetate	2.40	Disodium pamoate	0.14
Sodium hippurate	1.46	Disodium fumarate	0.725
Sodium potassium *L*-tartrate	1.40	Sodium propionate	5.20
Potassium antimony tartrate	0.054	Sodium propionate	2.60
Sodium diethyldithiocarbamate	0.011	Potassium *D*-saccharate	0.05
Disodium malonate	2.97	Sodium valerate	3.19
Sodium pyruvate	3.00	Trisodium phosphate	0.36
Sodium pyruvate	1.50	Disodium hydrogen phosphate	0.43
Sodium salicylate	2.20	Sodium dihydrogen phosphate	4.00
Sodium salicylate	1.10	Sodium dihydrogen phosphate	2.00
Sodium 2-ethylhexanoate	4.20	Sodium isethionate	2.20
Sodium 2-ethylhexanoate	2.10	Sodium isethionate	1.10
Sodium *L*-glutamate	2.00	Disodium oxalate	0.14
Sodium *L*-glutamate	1.00	Sodium hydrogen carbonate	0.60
Sodium sulfate	1.00	Sodium benzenesulfonate	1.10
Sodium tetraphenylborate	0.40	Sodium hexanoate	2.70
Sodium tetraphenylborate	0.20	Disodium carbonate	1.00
Potassium gluconate	1.10	Sodium adipate	1.19
Sodium saccharine	1.57	Sodium *L*-malate	2.92
Sodium 1-naphthalenacetate	0.43	Sodium dicyanamide	0.700
Sodium 1-naphthalensulfonate	0.35	Sodium nicotinate	2.96
Sodium 2-naphthalensulfonate	0.13	Sodium nicotinate	1.48
Disodium 2, 6-naphthalenedisulfonate	0.085	Sodium octanoate	1.56
Salt free			

Table S3 Crystallization results of ephedrinium chloride and alkaline iodide as a function of the ratio of iodide to ephedrinium, the cation of the iodide salt and the used silicone oil.

Silicone oil with 50 cSt viscosity

Molar ratio of iodide to ephedrinium	1	2.5	5	10	15	25	50	100	200
LiI				D3: needles[a]	D3: big needle	D3: needles[a]			
NaI				D3: needles[a]	D3: needles[a]	D3: big needle			
KI			D3: big needle	D3: needles[a]	D3: needles[a]	D5: needle[a]			

Silicone oil with 5 cSt viscosity

Molar ratio of iodide to ephedrinium	1	2.5	5	10	15	25	50	100	200
LiI			D3: needles[a]	D3: needles[a]	D3: big needle				
NaI			D3: needles[a]	D3: needles[a]	D3: plate	D3: needles[a]	D5: needle[a]		
KI			D3: needles[a]	D3: needles[a]	D3: needles[a]	D3: needles[a]			

Legend: DX: crystals were observed after X days. 100 µl of the premixed solutions were generated by pipetting appropriate volumes of a 1.15 M ephedrinium chloride and a 4 M alkaline iodide solution. 10 µl premixed solutions were placed at the bottom of 100 µl of the indicated silicone oil. [a]: well was scratched at day 3. All measured crystals belong to the orthorhombic polymorph I with a = 7.3 Å, b = 18.9 Å, c = 25.7 Å and a volume of 3562 Å^3 (Nievergelt *et al.*, 2018).

Table S4 Crystallization results of ephedrinium chloride and sodium oxalate as a function of the ratio of oxalate to ephedrinium and the used silicone oil.

Silicone oil with 50 cSt viscosity

Molar ratio of oxalate to ephedrinium	0.01	0.033	0.1	0.33	1	3.33	10	33.3	100
Na_2Oxalate	D1<br>D16: EphHCl	D1<br>D16: EphHCl	D1<br>D16: EphHCl	D1	D1	D1	D1	D16	

Silicone oil with 5 cSt viscosity

Molar ratio of oxalate to ephedrinium	0.01	0.033	0.1	0.33	1	3.33	10	33.3	100
Na_2Oxalate	D1<br>D16: EphHCl	D1<br>D16: EphHCl	D1<br>D16: EphHCl	D1	D1	D16			

Legend: DX: crystals were observed after X days. All observed crystals were bis ephedrinium oxalate (Nievergelt *et al.*, 2018), unless otherwise noted. 100 µl of the premixed solutions were generated by pipetting appropriate volumes of a 1.15 M ephedrinium chloride and a 0.14 M sodium oxalate. 10 µl of the premixed solutions were placed at the bottom of 100 µl of the indicated silicone oil. Additionally for the ratios of 0.1 and 1, solutions were directly mixed within the silicone oil to give a drop of 10 µl. The results were identical as for the corresponding premixed solutions.

S2. Crystallography

S2.1. [{*R*-carnitinenitrile}{*S*-carnitinenitrile}$_2$][chloride]$_3$

R,*S*-carnitinenitrile chloride surprisingly crystallized with three units of carnitinenitrile chloride (*S*-carnitinenitrile : *R*-carnitinenitrile ratio 2:1) in the asymmetric unit of the space group *P*2$_1$. Each hydroxy group hydrogen bonds to just one chloride ion, thereby forming ion pairs.

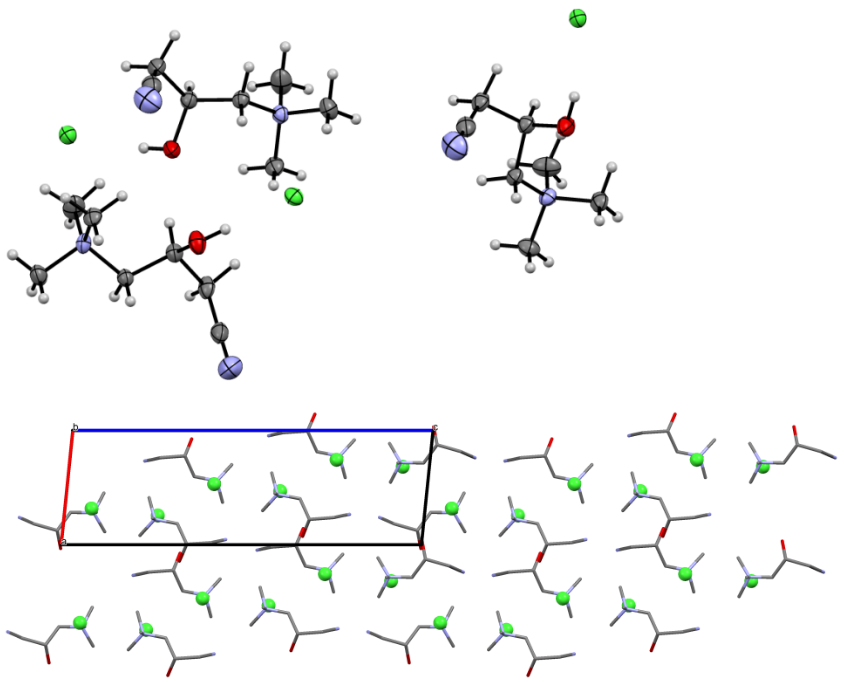

Figure S3 Top: displacement ellipsoid representation of [{(*R*)-Car}{(*S*)-Car}$_2$]·3Cl, ellipsoids are drawn at 50% probability. Below: packing diagram of [{(*R*)-Car}{(*S*)-Car}$_2$]·3Cl.

S2.2. [*R*,*S*-carnitinenitrile][iodide]

R,*S*-carnitinenitrile iodide crystallized with two formula units in the asymmetric unit of the space group *Pbca*. One of the carnitinenitrile cations has a disordered hydroxy group, as a result of both enantiomers randomly occupying the same crystallographic site. These hydroxy groups point to different iodide anions. There are weak hydrogen bonds between of the carnitinenitrile cation hydroxy groups and iodide anions.

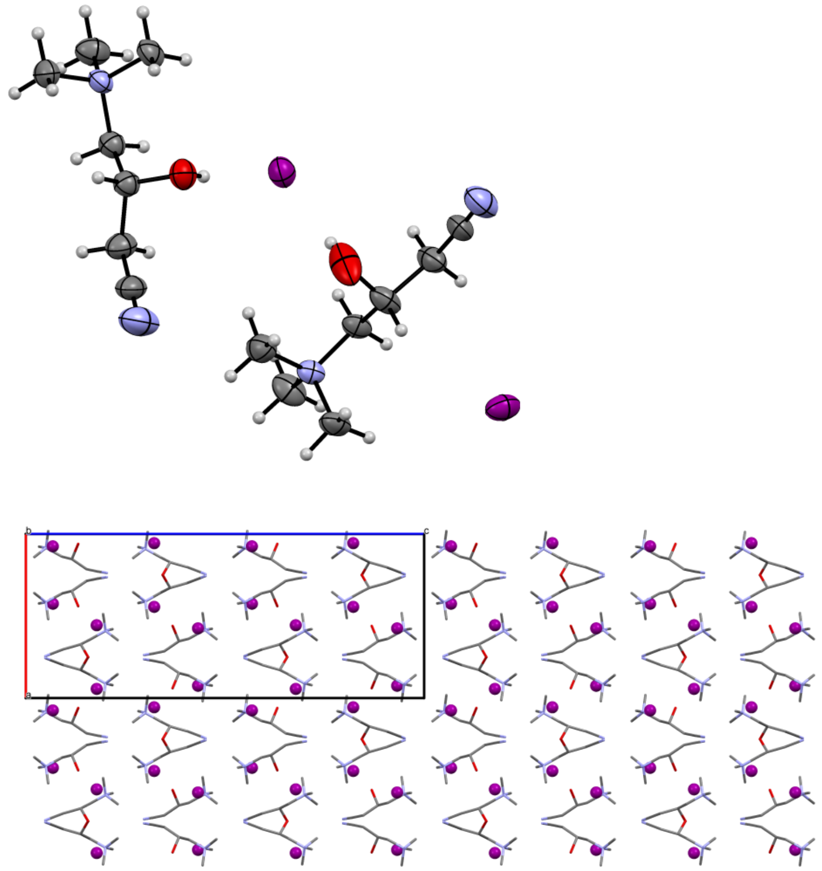

Figure S4 Top: displacement ellipsoid representation of [(+/−)-Car]I, ellipsoids are drawn at 50% probability. The disorder of the hydroxy group is omitted for clarity. Bottom: packing diagram of [(+/−)-Car]I.

S2.3. [*R*-carnitinenitrile][chloride]

R-carnitinenitrile chloride crystallized with one formula unit in the asymmetric unit of the chiral space group $P2_1$. Ion pairs result from a hydrogen bond between the chloride anion and the hydroxy group (O5) of the carnitinenitrile cation.

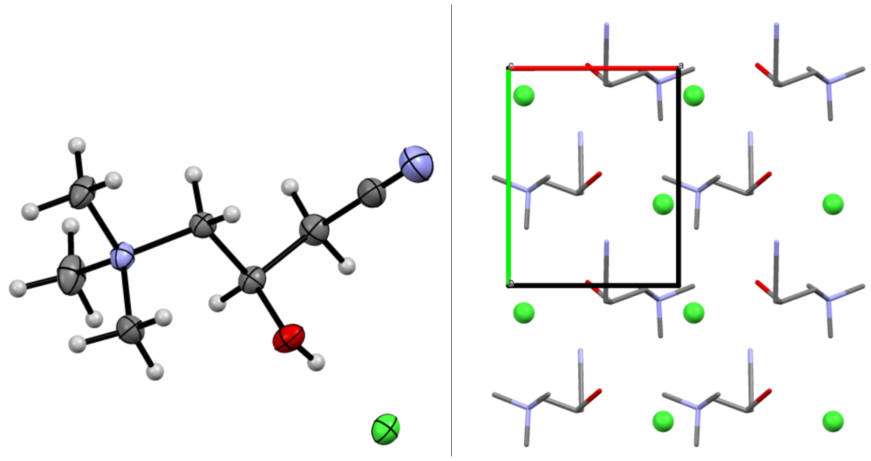

Figure S5 Left: displacement ellipsoid representation of [(−)-Car]Cl, ellipsoids are drawn at 50% probability. Right: packing diagram of [(−)-Car]Cl.

S2.4. [*R*-carnitinenitrile][bromide]

R-carnitinenitrile bromide crystallized with one formula unit in the asymmetric unit in the chiral space group $P2_1$. The structure is essentially isostructural with the chloride salt. One hydrogen bond between the bromide anion and the hydroxy group (O5) of the *R*-carnitinenitrile cation links the components into ion pairs.

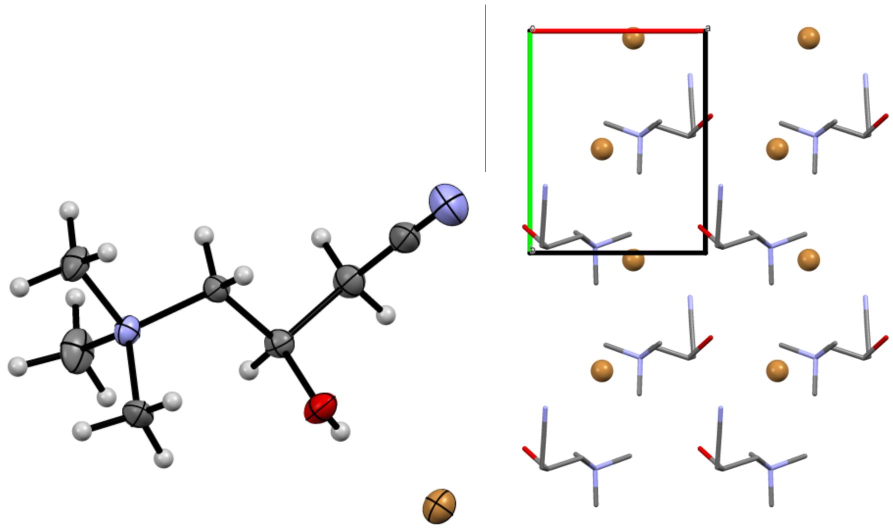

Figure S6 Left: displacement ellipsoid representation of [(−)-Car]Br, ellipsoids are drawn at 50% probability. Right: packing diagram of [(−)-Car]Br.

S2.5. [*R*-carnitinenitrile][iodide]

R-carnitinenitrile iodide crystallized with one formula unit in the asymmetric unit of the chiral space group $P2_12_12_1$. One hydrogen bond between the iodide anion and the hydroxyl group (O5) of the *R*-carnitinenitrile cation links the components into ion pairs.

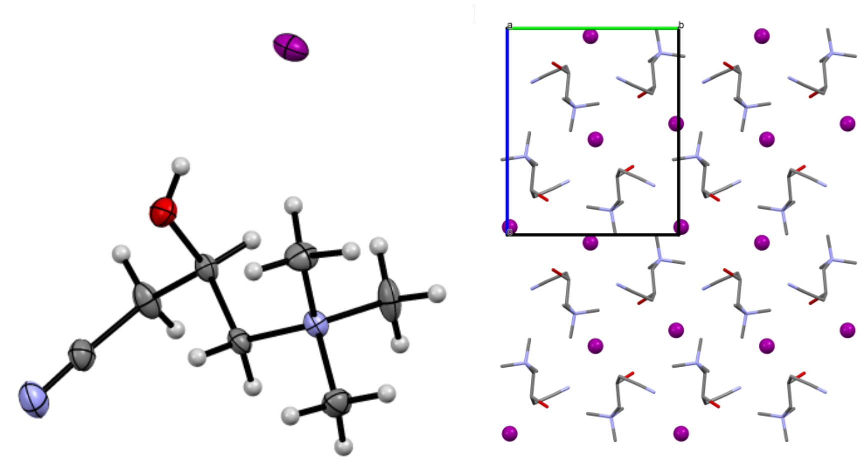

Figure S7 Left: displacement ellipsoid representation of [(−)-Car]I, ellipsoids are drawn at 50% probability. Right: packing diagram of [(−)-Car]I.

S2.6. [*R*-carnitinenitrile]$_2$[tetrafluoroborate][chloride]

Bis(*R*-carnitinenitrile)·tetrafluoroborate·chloride crystallized from a mixture of 5 µl of a 6.54 M *R*-carnitinenitrile chloride solution and 5 µl of a 4 M sodium tetrafluoroborate solution. The asymmetric unit of the space group $P2_12_12_1$ contains two *R*-carnitinenitrile cations, one chloride anion and one tetrafluoroborate anion. The tetrafluoroborate anions are disordered and they have no hydrogen bonding interaction. The displacement ellipsoids of the fluorine atoms were restrained to be similar during refinement. The structure is stabilized by two hydrogen bonds to the chloride anion from the hydroxy groups of two different *R*-carnitinenitrile cations. The chloride and tetrafluoroborate anions are stacked in an alternating fashion in the direction of the *c* axis. The dataset used for the structure determination had a relatively low completeness of 98.2 % to a Θ angle of 74.33°. This is caused by a small crystal and the use of symmetric kappa constraints to 1° and 60° for the strategy calculation in CrysAlisPro 1.171.39.12b. Unfortunately, all experiments to grow better single crystals have failed. On the other hand, the completeness of 98.2 % to the theta angle of 74.33° with the combination of low *R* indicators (0.0343; 0.0873 for all data) shows the accuracy of the result.

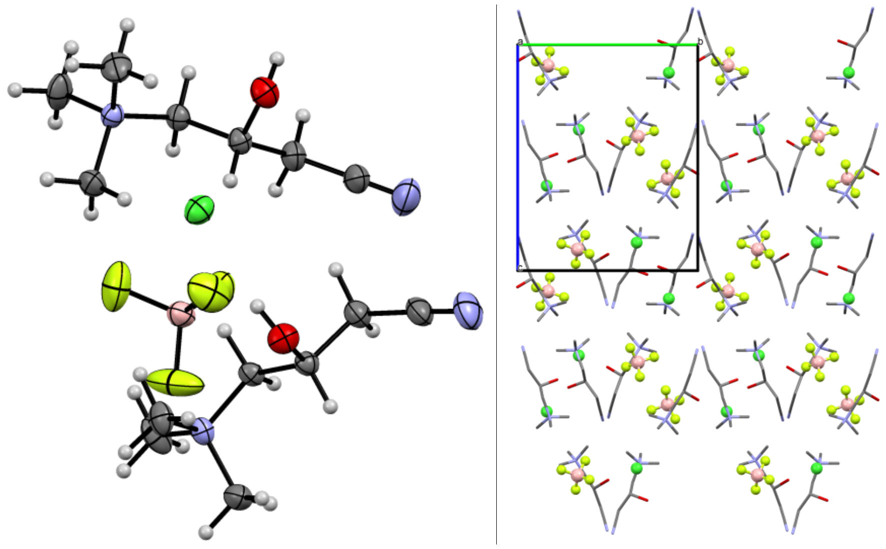

Figure S8 Left: displacement ellipsoid representation of [(−)-Car]$_2$[BF_4]Cl, ellipsoids are drawn at 50% probability. Right: packing diagram of [(−)-Car]$_2$[BF_4]Cl.

S2.7. [Diltiazem][bromide]

Diltiazem bromide crystallized with one formula unit in the asymmetric unit of the chiral space group $P2_12_12_1$. The structure is essentially isostructural with the published chloride salt (Kojic-Prodic *et al.*, 1984). During the refinement, it was necessary to use a different weighting scheme than normal. Instead of the modified Sheldrick weighting scheme, here a polynomial Chebychev weighting scheme was used (Prince, 1982, Watkin, 1994) since it gave lower R-values and lower shifts. One weak hydrogen bond between the bromide ion and the protonated tertiary amino group of diltiazem (N11) links the components into ion pairs. The distance between N11 and Br30 is 3.172(5) Å. However, there is another close intramolecular interaction between the keto group of the acetate fragment and the second keto group of diltiazem (O19···O15). The distance between these oxygen atoms is 2.943(7) Å.

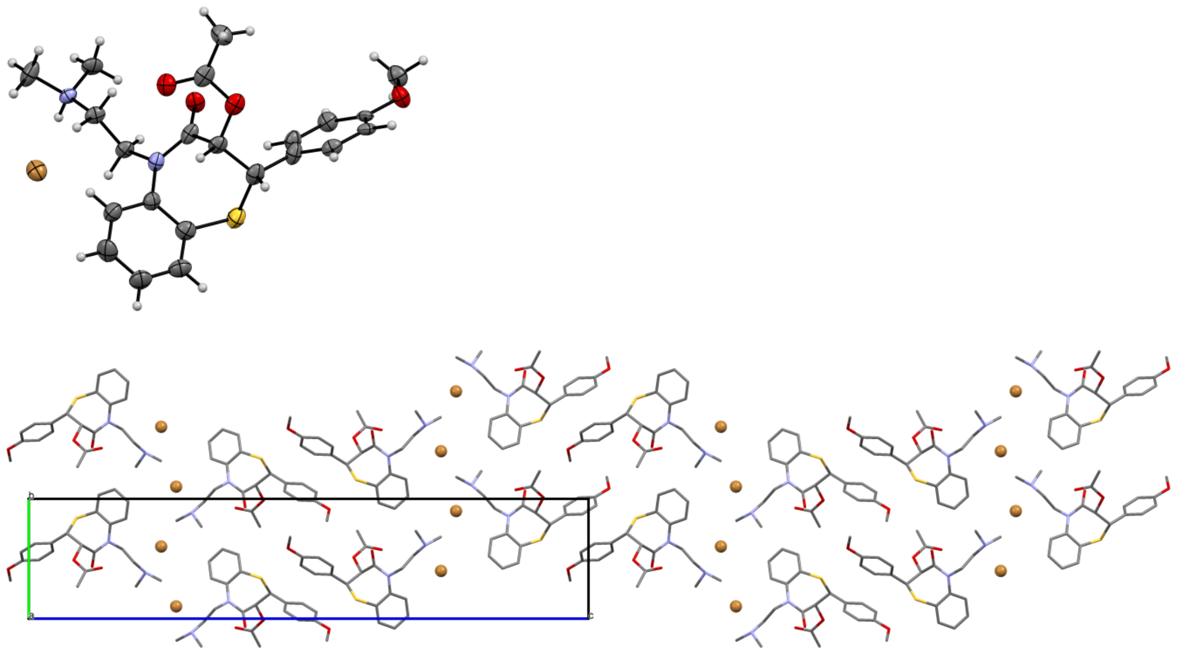

Figure S9 Top: displacement ellipsoid representation of [DilH]Br, ellipsoids are drawn at 50% probability. Below: packing diagram of [DilH]Br.

S2.8. [Diltiazem][iodide]

Diltiazem iodide crystallized with one formula unit in the asymmetric unit of the chiral space group $P2_12_12_1$. The structure is essentially isostructural with that of the published chloride salt (Kojic-Prodic *et al.*, 1984) and the aforementioned bromide salt. The crystal structure is stabilized by one weak hydrogen bond between iodide and the protonated tertiary amino group of diltiazem (N11). The distance between N11 and I30 is 3.3966(18) Å. However, there is another close intramolecular interaction between the keto group of the acetate fragment and the second keto group of diltiazem (O19···O15). The distance between the oxygen atoms is 2.978(3) Å.

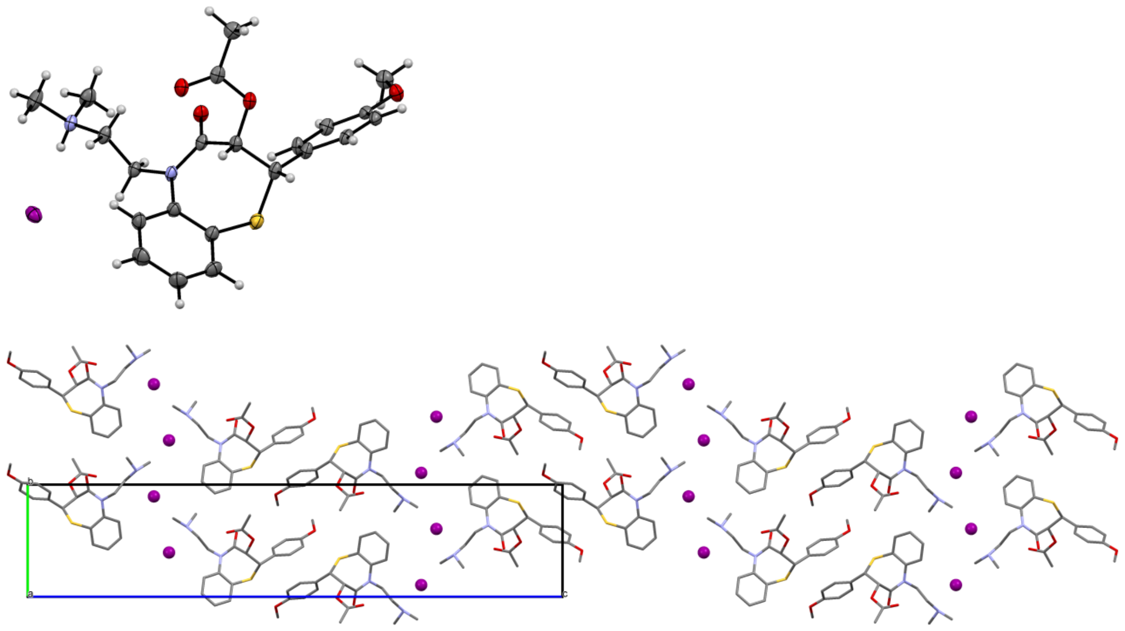

Figure S10 Top: displacement ellipsoid representation of [DilH]I, ellipsoids are drawn at 50% probability. Below: packing diagram of [DilH]I.

S2.9. [Diltiazem][nitrate]

Diltiazem nitrate crystallized with one formula unit in the asymmetric unit of the chiral space group $P2_12_12_1$. Interestingly, the crystal is essentially isomorphous with the published chloride salt (Kojic-Prodic *et al.*, 1984), as well as the aforementioned bromide and iodide salts. Ion pairs are formed by two hydrogen bonds between two oxygen atoms (O31; O32) of the nitrate anion and the protonated tertiary amino group of diltiazem (N11). Moreover, there is a close intramolecular interaction between the keto group of the acetate fragment and the second keto group of diltiazem (O19···O15). The distance between the oxygen atoms is 3.032(2) Å.

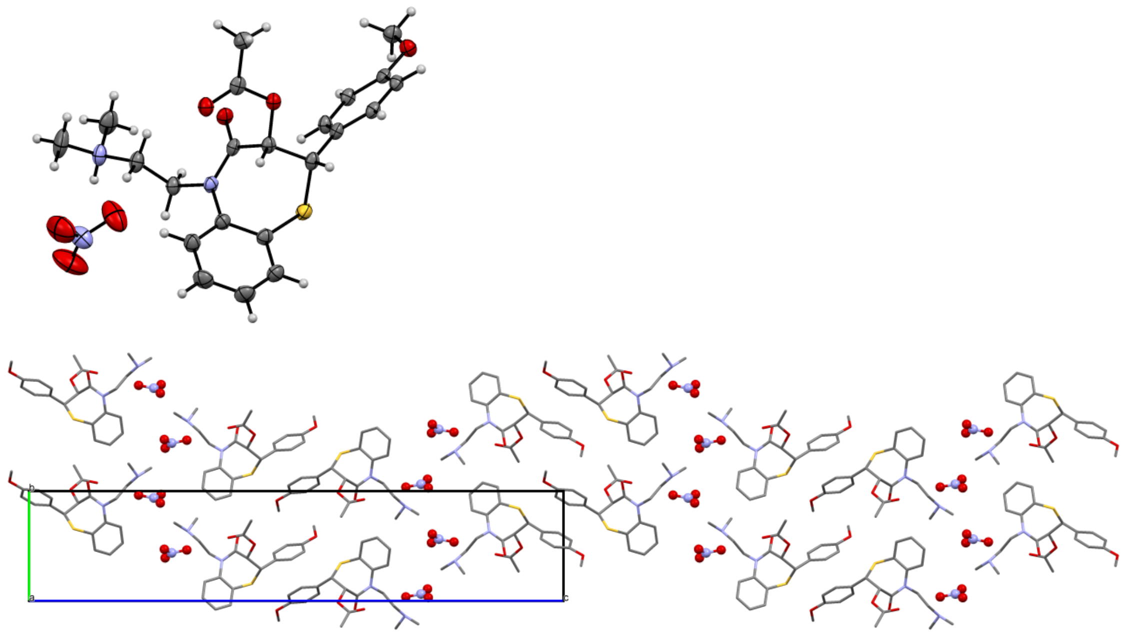

Figure S11 Top: displacement ellipsoid representation of [DilH][NO_3], ellipsoids are drawn at 50% probability. Below: packing diagram of [DilH][NO_3].

S2.10. [Diltiazem][dihydrogenphosphate]·1.5(H_2O)

Diltiazem dihydrogenphosphate crystallized with four units of diltiazem dihydrogenphosphate in the asymmetric unit of the chiral space group *P*1. The asymmetric unit also contains 6 molecules of water. There is a close intramolecular interaction between the keto group of the acetate moiety and the second keto group of all molecules of diltiazem (O19···O15) (3.021(4) Å 3.038(3) Å 3.035(3) Å 3.020(4) Å). CheckCIF reports a 90% match for the structure to one with a halved *b*-axis. Certainly, there is partial translation symmetry along *b*. However, it is obvious, that phosphate anions do not follow pseudo-translation. Even the side chains in (*b*/2)-related molecules of diltiazem differ significantly (see the bottom of Figure S12). Structure refinement in the halved-b unit cell does not reflect the real symmetry, and results in disorders. Hence, we preferred structure refinement in the supercell. It was possible to index about 70% of the reflections in a monoclinic cell with the dimensions: *a*: 8.79182(5) Å, *b*: 6.33050(4) Å, *c*: 46.6314(3) Å, *β*: 92.1049(5)°. The reflections could be nicely integrated by applying the space group *I*2 (R_{int} 6.5%) and the structure could be solved within that space group. However, that refinement did not yield a satisfying result as the position and geometry of the anion did not make any sense.

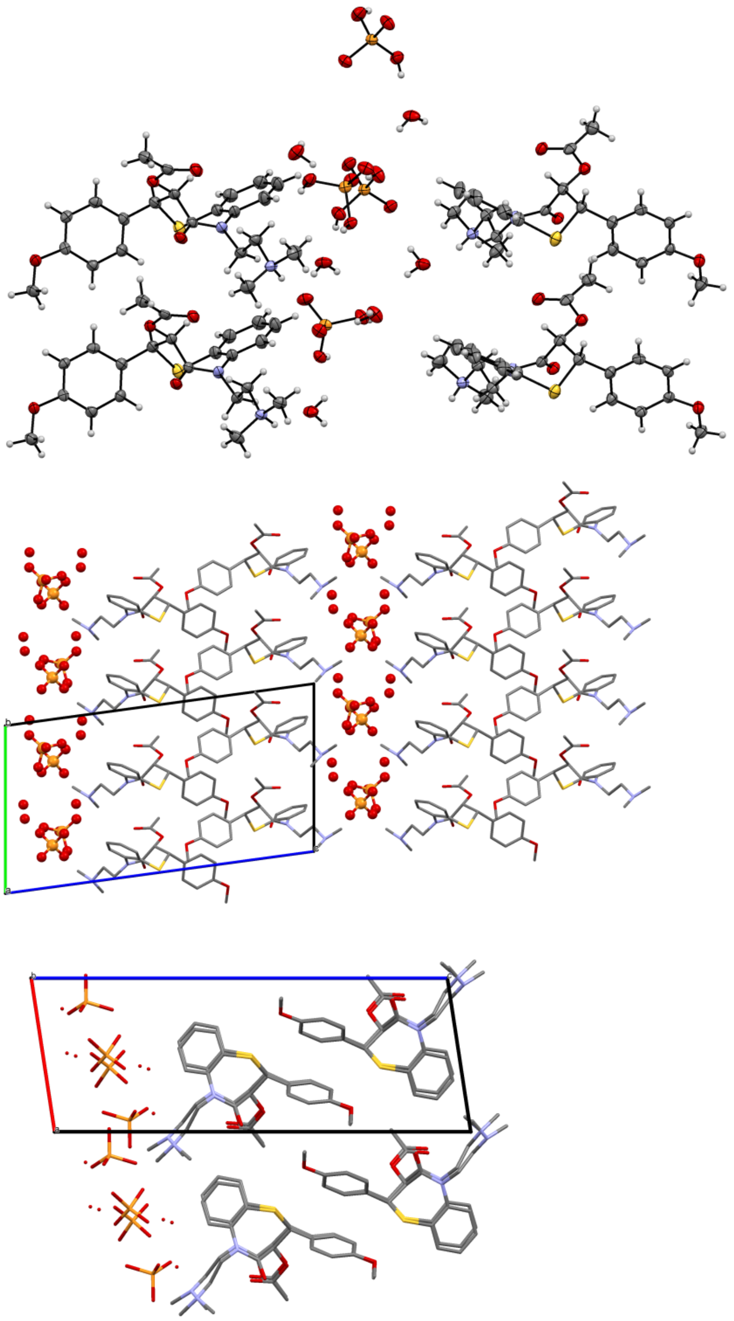

Figure S12 Top: displacement ellipsoid representation of $[DilH][H_2PO_4][H_2O]_{1.5}$, ellipsoids are drawn at 50% probability. Middle: packing diagram of $[DilH][H_2PO_4][H_2O]_{1.5}$. Bottom: Molecular packing of diltiazem dihydrogenphosphate, view along the *b* axis.

S2.11. [(1*S*,2*R*)-(+)-ephedrinium]$_2$[L-malate]·(H_2O)

Two cations of (1*S*,2*R*)-(+)-ephedrinium crystallized together with one L-malate anion and one water molecule in the asymmetric unit of the chiral space group *P*1.

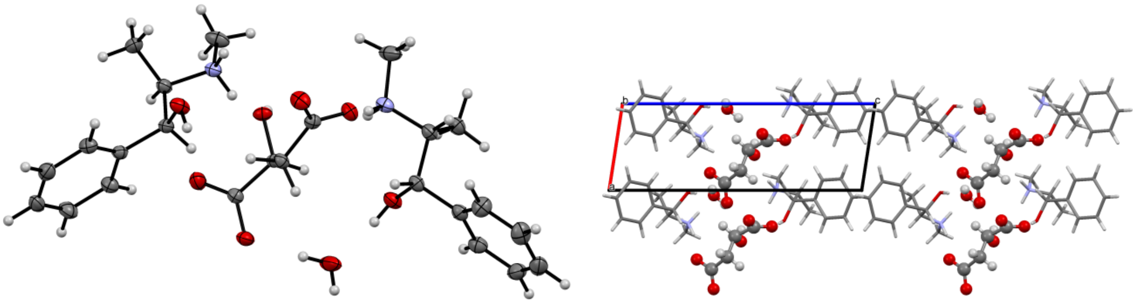

Figure S13 Left: displacement ellipsoid representation of [(1*S*,2*R*)-EphH]$_2$[L-malate](H_2O), ellipsoids are drawn at 50% probability. Right: packing diagram of [(1*S*,2*R*)-EphH]$_2$[L-malate](H_2O).

S2.12. [(1*S*,2*R*)-(+)-ephedrinium]$_2$[L-tartrate]·(H_2O)

Two cations of (1*S*,2*R*)-(+)-ephedrinium crystallized together with one L-tartrate anion and one water molecule in the asymmetric unit of the chiral space group *P*2$_1$.

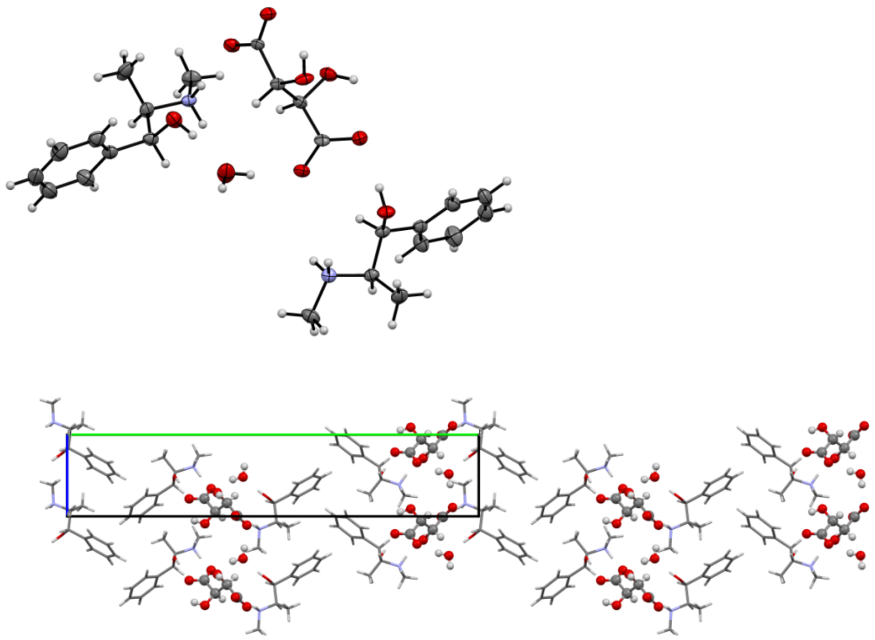

Figure S14 Top: displacement ellipsoid representation of [(1*S*,2*R*)-EphH]$_2$[L-tartrate](H_2O), ellipsoids are drawn at 50% probability. Below: packing diagram of [(1*S*,2*R*)-EphH]$_2$[L-tartrate](H_2O).

S2.13. [(1*S*,2*R*)-(+)-ephedrinium][nitrate], polymorph II

(1*S*,2*R*)-(+)-ephedrinium nitrate crystallized as thin plates with two formula units in the asymmetric unit of the chiral space group $P2_1$. The unit cell dimensions were a = 6.0401 (3) Å, b = 29.3553 (8) Å, c = 7.3828 (3) Å, β = 112.806 (5)° and V = 1206.70 (9) Å^3. This is a new polymorphic form of ephedrinium nitrate. Later, three-dimensional crystals (Figure S15) crystallized, which were identified to be the known nitrate salt, polymorph I, (Collier *et al.*, 2006) with unit cell dimensions of a = 5.536 (5) Å, 6.839 (9) Å, 15.669 (12) Å, β = 97.28 (7)° and V = 588(1) Å^3. As polymorph I was already known, only the initial unit cell constants were determined.

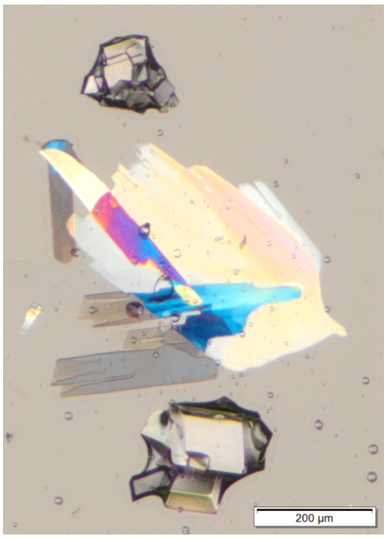

Figure S15 Microscope picture of (1*S*,2*R*)-(+)-ephedrinium nitrate crystals in Infinium oil: Polymorph I (three-dimensional crystals top and bottom) and polymorph II (thin plate-like crystals in the middle).

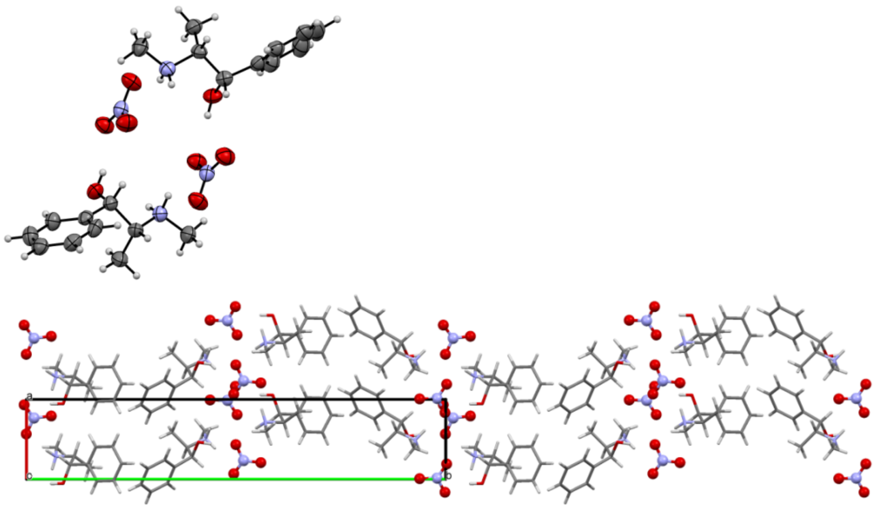

Figure S16 Top: displacement ellipsoid representation of (1*S*,2*R*)-(+)-ephedrinium nitrate, (polymorph II) ellipsoids are drawn at 50% probability. Below: packing diagram of (1*S*,2*R*)-(+)-ephedrinium nitrate (polymorph II).

S2.14. [(1*S*,2*R*)-(+)-ephedrinium][pyrrolidone carboxylate]·(H_2O)

(1*S*,2*R*)-(+)-ephedrinium pyrrolidone carboxylate crystallized in a 1:1 ratio together with one water molecule in the asymmetric unit of the chiral space group $P2_1$.

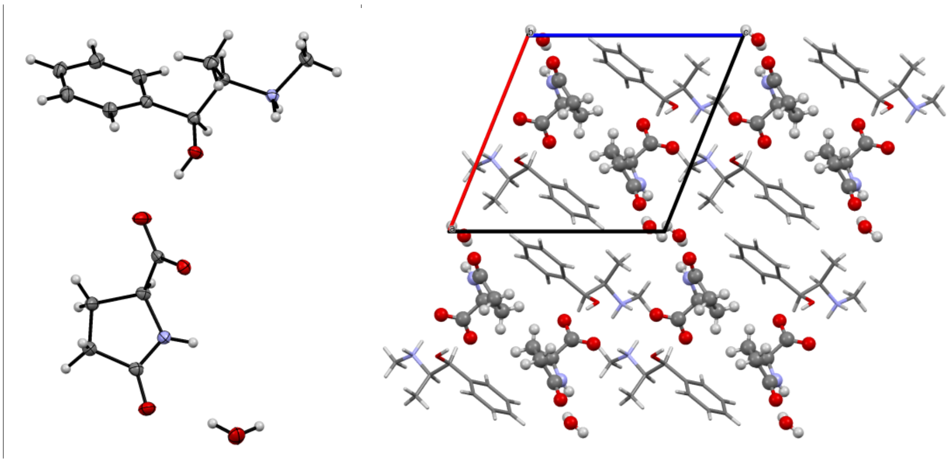

Figure S17 Left: displacement ellipsoid representation of [(1*S*,2*R*)-EphH][pyrrolidone carboxylate]·[H_2O], ellipsoids are drawn at 50% probability. Right: packing diagram of [(1*S*,2*R*)-EphH][pyrrolidone carboxylate]·[H_2O].

S2.15. [Trazodone][nitrate]

Trazodone nitrate crystallized with one formula unit in the asymmetric unit of the monoclinic space group $P2_1$/c.

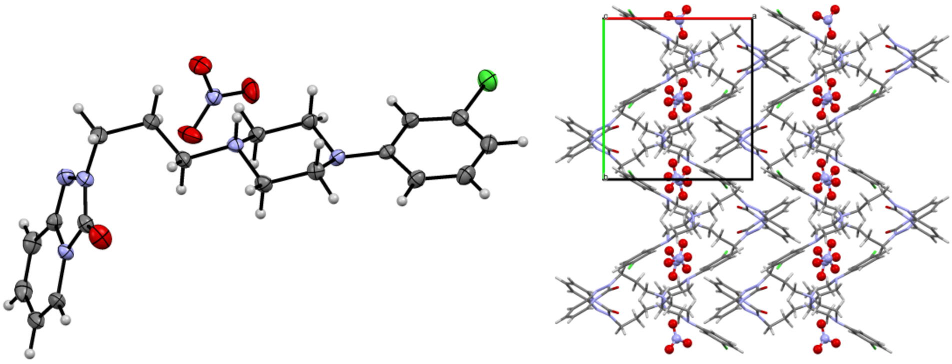

Figure S18 Left: displacement ellipsoid representation of [TrazH][NO_3], ellipsoids are drawn at 50% probability. Right: packing diagram of [TrazH][NO_3].

S2.16. [Trazodone][tetrafluoroborate]

Trazodone tetrafluoroborate crystallized with one formula unit in the asymmetric unit of the monoclinic space group $P2_1/c$. Three out of four fluorine atoms of the tetrafluoroborate anion are disordered in a 91:9 ratio.

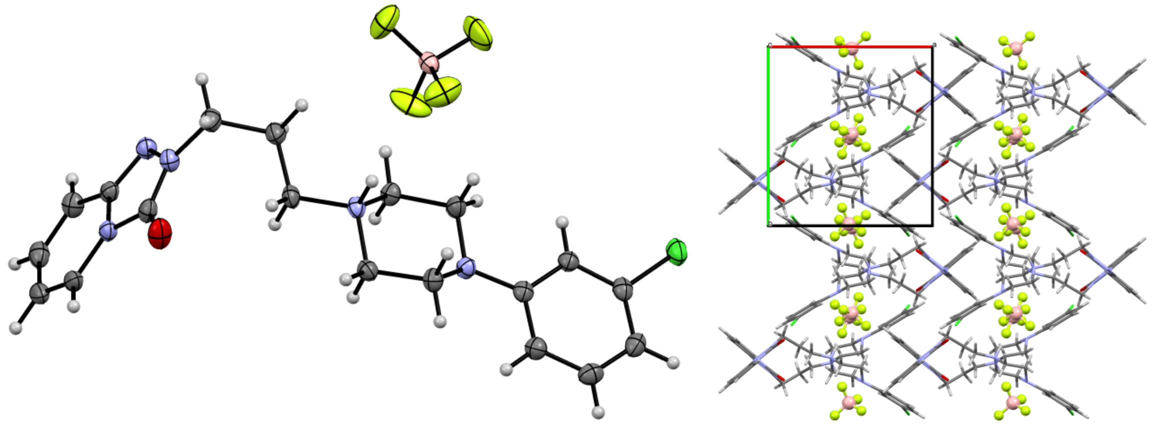

Figure S19 Left: displacement ellipsoid representation of [TrazH][BF_4], ellipsoids are drawn at 50% probability. The minor part of the disordered tetrafluoroborate was omitted for clarity. Right: packing diagram of [TrazH][BF_4].

S2.17. [Trazodone][thiocyanate]

Trazodone thiocyanate crystallized with two formula units in the asymmetric unit of the triclinic space group *P*-1. One thiocyanate is disordered in a 1:1 ratio, one trazodonium cation and the corresponding thiocyanate are disordered in a 51:49 ratio. Suitable restraints for distances and displacement ellipsoids (SADI, DFIX and RIGU) had to be applied.

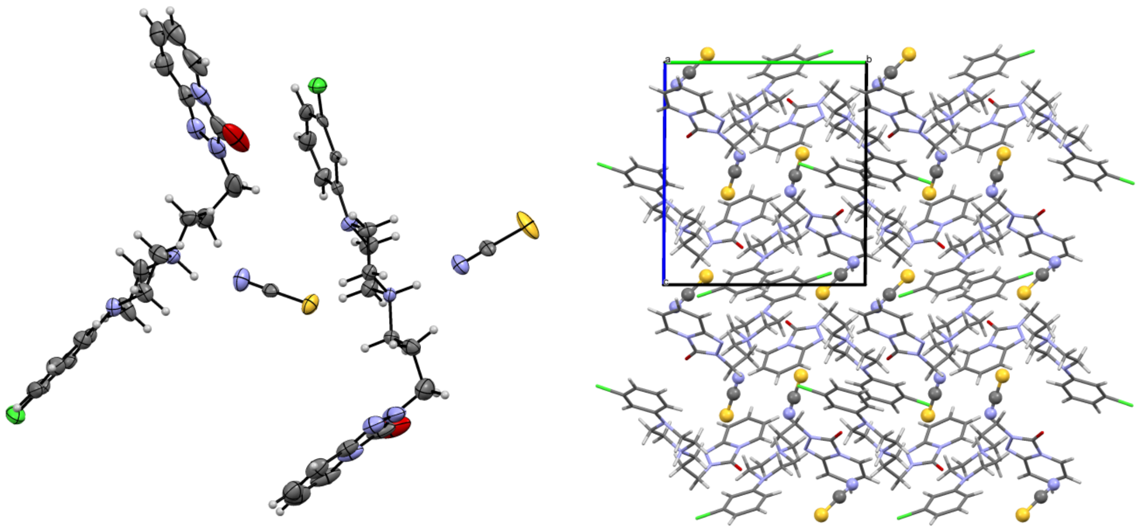

Figure S20 Left: displacement ellipsoid representation of [TrazH][SCN], ellipsoids are drawn at 50% probability. Only one moiety of both disordered thiocyanate anions and one disordered trazodonium cation is shown for clarity. Right: packing diagram of [TrazH][SCN], again not showing any disorder.

Table S5 Crystallographic information tables.

[Diltiazem][bromide]

Crystal data	
Chemical formula	$C_{22}H_{27}N_2O_4S \cdot Br$
M_r	495.44
Crystal system, space group	Orthorhombic, $P2_12_12_1$
Temperature (K)	160
a, b, c (Å)	6.1060 (3), 9.0317 (6), 42.330 (4)
V (Å^3)	2334.4 (3)
Z	4
Radiation type	Cu $K\alpha$
μ (mm^{-1})	3.48
Crystal shape	Plate
Crystal size (mm)	0.07 × 0.04 × 0.01
Data collection	
Diffractometer	XtaLAB Synergy, Dualflex, Pilatus 200K
Absorption correction	Multi-scan<br>*CrysAlis PRO*, (Rigaku Oxford Diffraction, 2015)
T_{min}, T_{max}	0.69, 0.95
No. of measured, independent and observed [$I > 2.0\sigma(I)$] reflections	24363, 4772, 4199
R_{int}	0.102
θ values (°)	$\theta_{max} = 74.5$, $\theta_{min} = 4.2$
$(\sin \theta/\lambda)_{max}$ (Å^{-1})	0.625
Refinement	
$R[F^2 > 2\sigma(F^2)]$, $wR(F^2)$, S	0.067, 0.092, 1.09
No. of reflections	4772
No. of parameters	272
H-atom treatment	H-atom parameters constrained
$\Delta\rho_{max}$, $\Delta\rho_{min}$ (e Å^{-3})	0.93, −0.72
Absolute structure	Flack (1983), 1977 Friedel-pairs
Absolute structure parameter	−0.04 (3)

Computer programs: *CrysAlis PRO*, (Rigaku Oxford Diffraction, 2015), Superflip (Palatinus & Chapuis, 2007), *CRYSTALS* (Betteridge *et al.*, 2003), *CAMERON* (Watkin *et al.*, 1996).

[Diltiazem][iodide]

Crystal data	
Chemical formula	$C_{22}H_{27}N_2O_4S{\cdot}I$
M_r	542.44
Crystal system, space group	Orthorhombic, $P2_12_12_1$
Temperature (K)	160
a, *b*, *c* (Å)	6.2219 (1), 9.0228 (1), 43.0569 (5)
V (Å^3)	2417.17 (5)
Z	4
Radiation type	Cu $K\alpha$
μ (mm^{-1})	11.46
Crystal shape	Prism
Crystal size (mm)	0.39 × 0.05 × 0.03
Data collection	
Diffractometer	XtaLAB Synergy, Dualflex, Pilatus 200K
Absorption correction	Multi-scan<br>*CrysAlis PRO*, (Rigaku Oxford Diffraction, 2015)
T_{min}, T_{max}	0.47, 0.70
No. of measured, independent and observed [$I > 2.0\sigma(I)$] reflections	15587, 4935, 4860
R_{int}	0.030
θ values (°)	$\theta_{max} = 74.5$, $\theta_{min} = 4.1$
$(\sin \theta/\lambda)_{max}$ (Å^{-1})	0.625
Refinement	
$R[F^2 > 2\sigma(F^2)]$, $wR(F^2)$, S	0.021, 0.053, 0.99
No. of reflections	4935
No. of parameters	272
H-atom treatment	H-atom parameters constrained
$\Delta\rho_{max}$, $\Delta\rho_{min}$ (e Å^{-3})	0.36, −0.37
Absolute structure	Flack (1983), 2050 Friedel-pairs
Absolute structure parameter	−0.013 (3)

Computer programs: *CrysAlis PRO*, (Rigaku Oxford Diffraction, 2015), *SIR92* (Altomare *et al.*, 1994), *CRYSTALS* (Betteridge *et al.*, 2003), *CAMERON* (Watkin *et al.*, 1996).

[Diltiazem][nitrate]

Crystal data	
Chemical formula	$C_{22}H_{27}N_2O_4S \cdot NO_3$
M_r	477.54
Crystal system, space group	Orthorhombic, $P2_12_12_1$
Temperature (K)	160
a, *b*, *c* (Å)	6.3765 (1), 8.8174 (1), 42.9420 (7)
V (Å^3)	2414.38 (6)
Z	4
Radiation type	Cu $K\alpha$
μ (mm^{-1})	1.59
Crystal shape	Prism
Crystal size (mm)	0.62 × 0.05 × 0.03
Data collection	
Diffractometer	XtaLAB Synergy, Dualflex, Pilatus 200K
Absorption correction	Multi-scan<br>*CrysAlis PRO*, (Rigaku Oxford Diffraction, 2015)
T_{min}, T_{max}	0.58, 0.96
No. of measured, independent and observed [$I > 2.0\sigma(I)$] reflections	25295, 4925, 4641
R_{int}	0.051
θ values (°)	$\theta_{max} = 74.5$, $\theta_{min} = 4.1$
$(\sin \theta/\lambda)_{max}$ (Å^{-1})	0.625
Refinement	
$R[F^2 > 2\sigma(F^2)]$, $wR(F^2)$, S	0.039, 0.093, 0.99
No. of reflections	4925
No. of parameters	299
H-atom treatment	H-atom parameters constrained
$\Delta\rho_{max}$, $\Delta\rho_{min}$ (e Å^{-3})	0.35, −0.26
Absolute structure	Flack (1983), 2051 Friedel-pairs
Absolute structure parameter	0.030 (18)

Computer programs: *CrysAlis PRO*, (Rigaku Oxford Diffraction, 2015), Superflip (Palatinus & Chapuis, 2007), *CRYSTALS* (Betteridge *et al.*, 2003), *CAMERON* (Watkin *et al.*, 1996).

[Diltiazem][dihydrogenphosphate](H_2O)$_{1.5}$

Crystal data	
Chemical formula	$(C_{22}H_{27}N_2O_4S)(H_2O_4P)(H_2O)_{1.5}$
M_r	539.53
Crystal system, space group	Triclinic, *P*1
Temperature (K)	160
a, *b*, *c* (Å)	8.7929 (1), 12.6645 (1), 23.7867 (2)
α, β, γ (°)	82.3345 (7), 81.4117 (7), 89.9824 (7)
V (Å^3)	2595.21 (4)
Z	4
Radiation type	Cu *K*α
μ (mm^{-1})	2.17
Crystal shape	Lath
Crystal size (mm)	0.17 × 0.07 × 0.02
Data collection	
Diffractometer	XtaLAB Synergy, Dualflex, Pilatus 200K
Absorption correction	Multi-scan *CrysAlis PRO*, (Rigaku Oxford Diffraction, 2015)
T_{min}, T_{max}	0.62, 0.96
No. of measured, independent and observed [$I > 2.0\sigma(I)$] reflections	264132, 19821, 18568
R_{int}	0.066
θ values (°)	θ_{max} = 72.1, θ_{min} = 3.5
$(\sin \theta/\lambda)_{max}$ (Å^{-1})	0.617
Refinement	
$R[F^2 > 2\sigma(F^2)]$, $wR(F^2)$, S	0.049, 0.128, 1.00
No. of reflections	19783
No. of parameters	1376
No. of restraints	66
H-atom treatment	H atoms treated by a mixture of independent and constrained refinement
$\Delta\rho_{max}$, $\Delta\rho_{min}$ (e Å^{-3})	1.49, −0.46
Absolute structure	Flack (1983), 9652 Friedel-pairs
Absolute structure parameter	0.020 (11)

Computer programs: *CrysAlis PRO*, (Rigaku Oxford Diffraction, 2015), Superflip (Palatinus & Chapuis, 2007), *CRYSTALS* (Betteridge *et al.*, 2003), *CAMERON* (Watkin *et al.*, 1996).

[*R*-Carnitinenitrile][chloride]

Crystal data	
Chemical formula	$C_7H_{15}N_2O{\cdot}Cl$
M_r	178.66
Crystal system, space group	Monoclinic, $P2_1$
Temperature (K)	160
a, *b*, *c* (Å)	7.2902 (2), 8.9099 (3), 7.8789 (3)
β (°)	107.024 (3)
V (Å^3)	489.35 (3)
Z	2
Radiation type	Mo $K\alpha$
μ (mm^{-1})	0.34
Crystal shape	Plate
Crystal size (mm)	0.30 × 0.20 × 0.07
Data collection	
Diffractometer	XtaLAB Synergy, Dualflex, Pilatus 200K
Absorption correction	Multi-scan *CrysAlis PRO*, (Rigaku Oxford Diffraction, 2015)
T_{min}, T_{max}	0.59, 0.98
No. of measured, independent and observed [$I > 2.0\sigma(I)$] reflections	15361, 3726, 3428
R_{int}	0.039
θ values (°)	θ_{max} = 33.1, θ_{min} = 2.7
$(\sin \theta/\lambda)_{max}$ (Å^{-1})	0.769
Refinement	
$R[F^2 > 2\sigma(F^2)]$, $wR(F^2)$, S	0.032, 0.081, 0.95
No. of reflections	3726
No. of parameters	101
No. of restraints	1
H-atom treatment	H-atom parameters not refined
$\Delta\rho_{max}$, $\Delta\rho_{min}$ (e Å^{-3})	0.39, −0.29
Absolute structure	Flack (1983), 1757 Friedel-pairs
Absolute structure parameter	−0.01 (4)

Computer programs: *CrysAlis PRO*, (Rigaku Oxford Diffraction, 2015), *SIR92* (Altomare *et al.*, 1994), *CRYSTALS* (Betteridge *et al.*, 2003), *CAMERON* (Watkin *et al.*, 1996).

[*R*-Carnitinenitrile][bromide]

Crystal data	
Chemical formula	$C_7H_{15}N_2O{\cdot}Br$
M_r	223.11
Crystal system, space group	Monoclinic, $P2_1$
Temperature (K)	160
a, b, c (Å)	7.5269 (2), 9.1017 (3), 7.9300 (3)
β (°)	107.161 (4)
V (Å^3)	519.08 (3)
Z	2
Radiation type	Mo $K\alpha$
μ (mm^{-1})	3.92
Crystal shape	Plate
Crystal size (mm)	0.12 × 0.10 × 0.03
Data collection	
Diffractometer	XtaLAB Synergy, Dualflex, Pilatus 200K
Absorption correction	Multi-scan<br>*CrysAlis PRO*, (Rigaku Oxford Diffraction, 2015)
T_{min}, T_{max}	0.64, 0.90
No. of measured, independent and observed [$I > 2.0\sigma(I)$] reflections	16508, 3402, 3118
R_{int}	0.034
θ values (°)	$\theta_{max} = 31.5$, $\theta_{min} = 2.7$
$(\sin \theta/\lambda)_{max}$ (Å^{-1})	0.735
Refinement	
$R[F^2 > 2\sigma(F^2)]$, $wR(F^2)$, S	0.029, 0.072, 0.97
No. of reflections	3402
No. of parameters	101
No. of restraints	1
H-atom treatment	H-atom parameters constrained
$\Delta\rho_{max}$, $\Delta\rho_{min}$ (e Å^{-3})	0.47, −0.53
Absolute structure	Flack (1983), 1581 Friedel-pairs
Absolute structure parameter	0.043 (10)

Computer programs: *CrysAlis PRO*, (Rigaku Oxford Diffraction, 2015), *SIR92* (Altomare *et al.*, 1994), *CRYSTALS* (Betteridge *et al.*, 2003), *CAMERON* (Watkin *et al.*, 1996).

[*R*-Carnitinenitrile][iodide]

Crystal data	
Chemical formula	$CC_7H_{15}N_2O{\cdot}I$
M_r	270.11
Crystal system, space group	Orthorhombic, $P2_12_12_1$
Temperature (K)	160
a, b, c (Å)	7.5850 (1), 10.8242 (2), 13.0468 (3)
V (Å^3)	1071.16 (3)
Z	4
Radiation type	Mo $K\alpha$
μ (mm^{-1})	2.95
Crystal shape	Prism
Crystal size (mm)	0.25 × 0.12 × 0.10
Data collection	
Diffractometer	XtaLAB Synergy, Dualflex, Pilatus 200K
Absorption correction	Multi-scan<br>*CrysAlis PRO*, (Rigaku Oxford Diffraction, 2015)
T_{min}, T_{max}	0.47, 0.75
No. of measured, independent and observed [$I > 2.0\sigma(I)$] reflections	17375, 3606, 3544
R_{int}	0.021
θ values (°)	$\theta_{max} = 32.9$, $\theta_{min} = 2.4$
$(\sin \theta/\lambda)_{max}$ (Å^{-1})	0.764
Refinement	
$R[F^2 > 2\sigma(F^2)]$, $wR(F^2)$, S	0.013, 0.030, 0.99
No. of reflections	3606
No. of parameters	101
H-atom treatment	H-atom parameters constrained
$\Delta\rho_{max}$, $\Delta\rho_{min}$ (e Å^{-3})	0.39, −0.41
Absolute structure	Flack (1983), 1481 Friedel-pairs
Absolute structure parameter	−0.018 (12)

Computer programs: *CrysAlis PRO*, (Rigaku Oxford Diffraction, 2015), Superflip (Palatinus & Chapuis, 2007), *CRYSTALS* (Betteridge *et al.*, 2003), *CAMERON* (Watkin *et al.*, 1996).

[*R*-Carnitinenitrile]$_2$[tetrafluoroborate][chloride]

Crystal data	
Chemical formula	$2(C_7H_{15}N_2O)\cdot BF_4\cdot Cl$
M_r	408.67
Crystal system, space group	Orthorhombic, $P2_12_12_1$
Temperature (K)	160
a, *b*, *c* (Å)	9.4006 (1), 13.2619 (2), 16.6581 (2)
V (Å^3)	2076.76 (5)
Z	4
Radiation type	Cu $K\alpha$
μ (mm^{-1})	2.09
Crystal shape	Block
Crystal size (mm)	0.13 × 0.07 × 0.05
Data collection	
Diffractometer	XtaLAB Synergy, Dualflex, Pilatus 200K
Absorption correction	Multi-scan<br>*CrysAlis PRO*, (Rigaku Oxford Diffraction, 2015)
T_{min}, T_{max}	0.75, 0.90
No. of measured, independent and observed [$I > 2.0\sigma(I)$] reflections	22105, 4175, 3958
R_{int}	0.037
θ values (°)	$\theta_{max} = 74.5$, $\theta_{min} = 4.3$
$(\sin \theta/\lambda)_{max}$ (Å^{-1})	0.625
Refinement	
$R[F^2 > 2\sigma(F^2)]$, $wR(F^2)$, S	0.033, 0.090, 1.00
No. of reflections	4175
No. of parameters	264
No. of restraints	19
H-atom treatment	H-atom parameters constrained
$\Delta\rho_{max}$, $\Delta\rho_{min}$ (e Å^{-3})	0.34, −0.19
Absolute structure	Flack (1983), 1797 Friedel-pairs
Absolute structure parameter	0.015 (13)

Computer programs: *CrysAlis PRO*, (Rigaku Oxford Diffraction, 2015), Superflip (Palatinus & Chapuis, 2007), *CRYSTALS* (Betteridge *et al.*, 2003), *CAMERON* (Watkin *et al.*, 1996).

[*R*,*S*-Carnitinenitrile][chloride]

Crystal data	
Chemical formula	$C_7H_{15}N_2O{\cdot}Cl$
M_r	178.66
Crystal system, space group	Monoclinic, $P2_1$
Temperature (K)	183
a, *b*, *c* (Å)	7.2588 (3), 8.9399 (4), 22.7927 (9)
β (°)	95.818 (4)
V (Å^3)	1471.47 (11)
Z	6
Radiation type	Mo $K\alpha$
μ (mm^{-1})	0.34
Crystal shape	Block
Crystal size (mm)	0.13 × 0.08 × 0.06
Data collection	
Diffractometer	XtaLAB Synergy, Dualflex, Pilatus 200K
Absorption correction	Multi-scan<br>*CrysAlis PRO*, (Rigaku Oxford Diffraction, 2015)
T_{min}, T_{max}	0.77, 0.98
No. of measured, independent and observed [$I > 2.0\sigma(I)$] reflections	23769, 10401, 7769
R_{int}	0.032
θ values (°)	$\theta_{max} = 32.6$, $\theta_{min} = 2.5$
$(\sin \theta/\lambda)_{max}$ (Å^{-1})	0.758
Refinement	
$R[F^2 > 2\sigma(F^2)]$, $wR(F^2)$, S	0.052, 0.128, 1.00
No. of reflections	10401
No. of parameters	299
No. of restraints	1
H-atom treatment	H-atom parameters constrained
$\Delta\rho_{max}$, $\Delta\rho_{min}$ (e Å^{-3})	0.75, −0.43
Absolute structure	Flack (1983), 4756 Friedel-pairs
Absolute structure parameter	−0.03 (4)

Computer programs: *CrysAlis PRO*, (Rigaku Oxford Diffraction, 2015), Superflip (Palatinus & Chapuis, 2007), *CRYSTALS* (Betteridge *et al.*, 2003), *CAMERON* (Watkin *et al.*, 1996).

[*R*,*S*-Carnitinenitrile][iodide]

Crystal data	
Chemical formula	$C_7H_{15}N_2O{\cdot}I$
M_r	270.11
Crystal system, space group	Orthorhombic, *Pbca*
Temperature (K)	160
a, *b*, *c* (Å)	13.4725 (2), 9.8551 (1), 32.7259 (4)
V (Å^3)	4345.11 (9)
Z	16
Radiation type	Cu $K\alpha$
μ (mm^{-1})	22.83
Crystal shape	Plate
Crystal size (mm)	0.12 × 0.10 × 0.05
Data collection	
Diffractometer	XtaLAB Synergy, Dualflex, Pilatus 200K
Absorption correction	Multi-scan<br>*CrysAlis PRO* (Rigaku Oxford Diffraction, 2017)
T_{min}, T_{max}	0.12, 0.34
No. of measured, independent and observed [$I > 2.0\sigma(I)$] reflections	17435, 4437, 4082
R_{int}	0.032
θ values (°)	$\theta_{max} = 74.5$, $\theta_{min} = 4.3$
$(\sin \theta/\lambda)_{max}$ (Å^{-1})	0.625
Refinement	
$R[F^2 > 2\sigma(F^2)]$, $wR(F^2)$, S	0.049, 0.113, 1.01
No. of reflections	4437
No. of parameters	209
No. of restraints	2
H-atom treatment	H-atom parameters constrained
	Method = Modified Sheldrick $w = 1/[\sigma^2(F^2) + (0.03P)^2 + 31.74P]$,<br>where $P = (\max(F_o^2,0) + 2F_c^2)/3$
$\Delta\rho_{max}$, $\Delta\rho_{min}$ (e Å^{-3})	1.97, −1.61

Computer programs: *CrysAlis PRO* (Rigaku Oxford Diffraction, 2017), Superflip (Palatinus & Chapuis, 2007), *CRYSTALS* (Betteridge *et al.*, 2003), *CAMERON* (Watkin *et al.*, 1996).

[(1*S*,2*R*)-(+)-ephedrinium]$_2$[L-malate]·(H_2O)

Crystal data	
Chemical formula	$2(C_{10}H_{16}NO)\cdot C_4H_4O_5\cdot H_2O$
M_r	482.56
Crystal system, space group	Triclinic, *P*1
Temperature (K)	160
a, *b*, *c* (Å)	5.82130 (7), 7.27088 (8), 15.90214 (13)
α, β, γ (°)	94.7215 (8), 96.6693 (8), 109.5246 (11)
V (Å^3)	624.78 (1)
Z	1
Radiation type	Cu *K*α
μ (mm^{-1})	0.79
Crystal size (mm)	0.3 × 0.21 × 0.01
Data collection	
Diffractometer	XtaLAB Synergy, Dualflex, Pilatus 200K
Absorption correction	Gaussian<br>*CrysAlis PRO* 1.171.40.16c (Rigaku Oxford Diffraction, 2018) Numerical absorption follwed by empirical absorption correction.
T_{min}, T_{max}	0.463, 1.000
No. of measured, independent and observed [$I > 2\sigma(I)$] reflections	19924, 4803, 4709
R_{int}	0.032
$(\sin \theta/\lambda)_{max}$ (Å^{-1})	0.636
Refinement	
$R[F^2 > 2\sigma(F^2)]$, $wR(F^2)$, S	0.027, 0.074, 1.06
No. of reflections	4803
No. of parameters	322
No. of restraints	3
H-atom treatment	mixed
$\Delta\rho_{max}$, $\Delta\rho_{min}$ (e Å^{-3})	0.16, −0.18
Absolute structure	Flack x determined using 2188 quotients [(I+)-(I-)]/[(I+)+(I-)] (Parsons, Flack and Wagner, Acta Cryst. B69 (2013) 249-259).
Abs. struct. parameter	0.00 (6)

Computer programs: *CrysAlis PRO* 1.171.40.16c (Rigaku OD, 2018), *SHELXL* (Sheldrick, 2015), Olex2 (Dolomanov *et al.*, 2009).

[(1*S*,2*R*)-(+)-ephedrinium][nitrate],·polymorph II

Crystal data	
Chemical formula	$C_{10}H_{16}NO\cdot NO_3$
M_r	228.25
Crystal system, space group	Monoclinic, $P2_1$
Temperature (K)	160
a, *b*, *c* (Å)	6.0401 (3), 29.3553 (8), 7.3828 (3)
β (°)	112.806 (5)
V (Å^3)	1206.70 (9)
Z	4
Radiation type	Cu $K\alpha$
μ (mm^{-1})	0.82
Crystal size (mm)	0.40 × 0.07 × 0.01
Data collection	
Diffractometer	XtaLAB Synergy, Dualflex, Pilatus 200K
Absorption correction	*CrysAlis PRO* 1.171.39.46 (Rigaku Oxford Diffraction, 2018) Numerical absorption followed by empirical absorption correction.
T_{min}, T_{max}	0.811, 1.000
No. of measured, independent and observed [$I > 2\sigma(I)$] reflections	12312, 4637, 3727
R_{int}	0.046
$(\sin \theta/\lambda)_{max}$ (Å^{-1})	0.632
Refinement	
$R[F^2 > 2\sigma(F^2)]$, $wR(F^2)$, S	0.068, 0.190, 1.09
No. of reflections	4637
No. of parameters	295
No. of restraints	1
H-atom treatment	H-atom parameters constrained
$\Delta\rho_{max}$, $\Delta\rho_{min}$ (e Å^{-3})	0.63, −0.30
Absolute structure	Flack x determined using 1465 quotients [(I+)-(I-)]/[(I+)+(I-)] (Parsons, Flack and Wagner, Acta Cryst. B69 (2013) 249-259).
Abs. struct. parameter	0.1 (2)

Computer programs: *CrysAlis PRO* 1.171.39.46 (Rigaku OD, 2018), *SHELXL* (Sheldrick, 2015), Olex2 (Dolomanov *et al.*, 2009).

[(1*S*,2*R*)-(+)-ephedrinium][pyrrolidone carboxylate]·(H_2O)

Crystal data	
Chemical formula	$C_{10}H_{16}NO{\bullet}C_5H_6NO_3{\bullet}H_2O$
Mr	312.36
Crystal system, space group	Monoclinic, $P2_1$
Temperature (K)	160
a, b, c (Å)	11.47377 (11), 6.14747 (5), 11.71348 (12)
β (°)	111.9780 (11)
V (Å^3)	766.16 (1)
Z	2
Radiation type	Cu *Kα*
μ (mm^{-1})	0.84
Crystal size (mm)	0.38 × 0.04 × 0.03
Data collection	
Diffractometer	XtaLAB Synergy, Dualflex, Pilatus 200K
Absorption correction	Gaussian<br>CrysAlis PRO 1.171.39.46 (Rigaku Oxford Diffraction, 2018) Numerical absorption correction based on gaussian integration over a multifaceted crystal model Empirical absorption correction using spherical harmonics, implemented in SCALE3 ABSPACK scaling algorithm.
Tmin, Tmax	0.708, 1.000
No. of measured, independent and observed [I > 2σ(I)] reflections	13893, 3268, 3223
Rint	0.024
$(\sin \theta/\lambda)_{max}$ (Å^{-1})	0.636
Refinement	
$R[F^2 > 2\sigma(F^2)]$, $wR(F^2)$, S	0.025, 0.066, 1.04
No. of reflections	3268
No. of parameters	205
No. of restraints	1
H-atom treatment	H-atom parameters constrained
Δρmax, Δρmin (e Å^{-3})	0.20, −0.14
Absolute structure	Flack x determined using 1425 quotients [(I+)-(I-)]/[(I+)+(I-)] (Parsons, Flack and Wagner, Acta Cryst. B69 (2013) 249-259).
Absolute structure parameter	0.03 (5)

Computer programs: *CrysAlis PRO* 1.171.39.46 (Rigaku OD, 2018), *SHELXL* (Sheldrick, 2015), Olex2 (Dolomanov *et al.*, 2009).

[(1*S*,2*R*)-(+)-ephedrinium]$_2$[L-tartrate]·(H_2O)

Crystal data	
Chemical formula	$2(C_{10}H_{16}NO)\cdot C_4H_4O_6\cdot H_2O$
M_r	498.56
Crystal system, space group	Monoclinic, $P2_1$
Temperature (K)	160
a, *b*, *c* (Å)	6.07189 (12), 32.9414 (4), 7.1383 (1)
β (°)	114.168 (2)
V (Å^3)	1302.63 (4)
Z	2
Radiation type	Cu $K\alpha$
μ (mm^{-1})	0.81
Crystal size (mm)	0.48 × 0.35 × 0.01
Data collection	
Diffractometer	XtaLAB Synergy, Dualflex, Pilatus 200K
Absorption correction	*CrysAlis PRO* 1.171.39.46 (Rigaku Oxford Diffraction, 2018) Numerical absorption followed by empirical absorption correction.
T_{min}, T_{max}	0.370, 1.000
No. of measured, independent and observed [$I > 2\sigma(I)$] reflections	31569, 5216, 5091
R_{int}	0.047
$(\sin \theta/\lambda)_{max}$ (Å^{-1})	0.636
Refinement	
$R[F^2 > 2\sigma(F^2)]$, $wR(F^2)$, S	0.042, 0.111, 1.10
No. of reflections	5216
No. of parameters	329
No. of restraints	2
H-atom treatment	H atoms treated by a mixture of independent and constrained refinement
$\Delta\rho_{max}$, $\Delta\rho_{min}$ (e Å^{-3})	0.34, −0.29
Absolute structure	Flack x determined using 2347 quotients [(I+)-(I-)]/[(I+)+(I-)] (Parsons, Flack and Wagner, Acta Cryst. B69 (2013) 249-259).
Abs. struct. parameter	0.00 (7)

Computer programs: *CrysAlis PRO* 1.171.39.46 (Rigaku OD, 2018), *SHELXL* (Sheldrick, 2015), Olex2 (Dolomanov *et al.*, 2009).

[Trazodonium][nitrate]

Crystal data	
Chemical formula	$[C_{19}H_{23}ClN_5O][NO_3]$
M_r	434.88
Crystal system, space group	Monoclinic, $P2_1/c$
Temperature (K)	160
a, *b*, *c* (Å)	12.6065 (3), 13.0726 (3), 12.6694 (3)
β (°)	106.397 (3)
V (Å^3)	2003.00 (8)
Z	4
Radiation type	Mo *K*α
μ (mm^{-1})	0.23
Crystal size (mm)	0.53 × 0.20 × 0.17
Data collection	
Diffractometer	XtaLAB Synergy, Dualflex, Pilatus 200K
Absorption correction	Gaussian<br>*CrysAlis PRO* 1.171.39.46 (Rigaku Oxford Diffraction, 2018) Numerical absorption correction based on gaussian integration over a multifaceted crystal model Empirical absorption correction using spherical harmonics, implemented in SCALE3 ABSPACK scaling algorithm.
T_{min}, T_{max}	0.560, 1.000
No. of measured, independent and observed [$I > 2\sigma(I)$] reflections	21495, 6532, 5530
R_{int}	0.020
$(\sin \theta/\lambda)_{max}$ (Å^{-1})	0.771
Refinement	
$R[F^2 > 2\sigma(F^2)]$, $wR(F^2)$, S	0.036, 0.106, 1.05
No. of reflections	6532
No. of parameters	274
H-atom treatment	H atoms treated by a mixture of independent and constrained refinement
$\Delta\rho_{max}$, $\Delta\rho_{min}$ (e Å^{-3})	0.40, −0.29

Computer programs: *CrysAlis PRO* 1.171.39.46 (Rigaku OD, 2018), *SHELXT* (Sheldrick, 2015), *SHELXL* (Sheldrick, 2015), Olex2 (Dolomanov *et al.*, 2009).

[Trazodonium][tetrafluoroborate]

Crystal data	
Chemical formula	$[C_{19}H_{23}ClN_5O][BF_4]$
M_r	459.68
Crystal system, space group	Monoclinic, $P2_1/c$
Temperature (K)	160
a, *b*, *c* (Å)	12.46473 (13), 12.98315 (16), 13.42014 (13)
β (°)	106.7648 (11)
V (Å^3)	2079.49 (4)
Z	4
Radiation type	Cu $K\alpha$
μ (mm^{-1})	2.15
Crystal size (mm)	0.19 × 0.17 × 0.02
Data collection	
Diffractometer	XtaLAB Synergy, Dualflex, Pilatus 200K
Absorption correction	Gaussian *CrysAlis PRO* 1.171.39.46 (Rigaku Oxford Diffraction, 2018) Numerical absorption correction based on gaussian integration over a multifaceted crystal model Empirical absorption correction using spherical harmonics, implemented in SCALE3 ABSPACK scaling algorithm.
T_{min}, T_{max}	0.531, 1.000
No. of measured, independent and observed [$I > 2\sigma(I)$] reflections	48534, 4315, 4165
R_{int}	0.029
$(\sin \theta/\lambda)_{max}$ (Å^{-1})	0.636
Refinement	
$R[F^2 > 2\sigma(F^2)]$, $wR(F^2)$, S	0.042, 0.104, 1.16
No. of reflections	4315
No. of parameters	293
H-atom treatment	H-atom parameters constrained
$\Delta\rho_{max}$, $\Delta\rho_{min}$ (e Å^{-3})	0.33, −0.31

Computer programs: *CrysAlis PRO* 1.171.39.46 (Rigaku OD, 2018), *SHELXT* (Sheldrick, 2015), *SHELXL* (Sheldrick, 2015), Olex2 (Dolomanov *et al.*, 2009).

[Trazodonium][thiocyanate]

Crystal data	
Chemical formula	$[C_{19}H_{23}ClN_5O][CNS]$
M_r	430.95
Crystal system, space group	Triclinic, *P*1
Temperature (K)	160
a, *b*, *c* (Å)	12.1482 (2), 13.1848 (2), 13.82510 (17)
α, β, γ (°)	90.2630 (12), 90.2180 (12), 108.0045 (15)
V ($Å^3$)	2105.89 (6)
Z	4
Radiation type	Cu *K*α
μ (mm^{-1})	2.73
Crystal size (mm)	0.23 × 0.19 × 0.07
Data collection	
Diffractometer	XtaLAB Synergy, Dualflex, Pilatus 200K
Absorption correction	Gaussian<br>*CrysAlis PRO* 1.171.39.46 (Rigaku Oxford Diffraction, 2018) Numerical absorption correction based on gaussian integration over a multifaceted crystal model Empirical absorption correction using spherical harmonics, implemented in SCALE3 ABSPACK scaling algorithm.
T_{min}, T_{max}	0.532, 1.000
No. of measured, independent and observed [$I > 2\sigma(I)$] reflections	52525, 8412, 7824
R_{int}	0.032
$(\sin \theta/\lambda)_{max}$ ($Å^{-1}$)	0.636
Refinement	
$R[F^2 > 2\sigma(F^2)]$, $wR(F^2)$, S	0.057, 0.136, 1.05
No. of reflections	8412
No. of parameters	723
No. of restraints	45
H-atom treatment	H-atom parameters constrained
$\Delta\rho_{max}$, $\Delta\rho_{min}$ (e $Å^{-3}$)	0.51, −0.55

Computer programs: *CrysAlis PRO* 1.171.39.46 (Rigaku OD, 2018), *SHELXT* (Sheldrick, 2015), *SHELXL* (Sheldrick, 2015), Olex2 (Dolomanov *et al.*, 2009).

References

Collier, E. A., Davey, R. J., Black, S. N. & Roberts, R. J. (2006). *Acta Cryst.* **B62**, 498-505.
Kojic-Prodic, B., Ruzic-Toros, Z., Sunjic, V., Decorte, E. & Moimas, F. (1984). *Helv. Chim. Acta* **67**, 916-926.
Nievergelt, P. P., Babor, M., Cejka, J. & Spingler, B. (2018). *Chem. Sci.* **9**, 3716-3722.
Prince, E. (1982). *Mathematical techniques in crystallography and materials science*. New York: Springer.
Watkin, D. (1994). *Acta Cryst.* **A50**, 411-437.
